# Automated Identification of Thermal Transitions in Conjugated Material Thin Films Using In Situ Optical Spectroscopy

**DOI:** 10.1002/smtd.202502209

**Published:** 2026-03-31

**Authors:** Doan Vu, Martyn Jevric, Alireza Samadani, Mats R. Andersson, Christopher R. McNeill, Brendan T. O'Connor, Harald Ade

**Affiliations:** ^1^ Department of Physics and Organic and Carbon Electronics Laboratories (ORaCEL) North Carolina State University Raleigh North Carolina USA; ^2^ Department of Mechanical and Aerospace Engineering and ORaCEL North Carolina State University Raleigh North Carolina USA; ^3^ Flinders Institute for Nanoscale Science and Technology Flinders University Adelaide South Australia Australia; ^4^ Department of Materials Science and Engineering Monash University Clayton Victoria Australia

**Keywords:** automated analysis, conjugated materials, thermal transitions, thin films, UV–vis spectroscopy, ATLAS

## Abstract

The thermal transitions of organic semiconductors have important implications for morphological and thermomechanical properties of electronic devices. Here we show that in situ UV–vis spectroscopy coupled with automated data analysis is a facile tool to determine the numerous thermal transitions that can exist in organic semiconductor thin films. Thermal transitions are automatically identified through employing linear segment detection of the semilogarithmic normalized deviation metric (NDM) as well as the spectral changes using piecewise linear regression. Furthermore, by comparing the in situ UV–vis with other thermal measurement methods we observe the temperature dependent absorbance can be used to discern the type of thermal transitions including liquid crystal transitions, glass transitions, and melting temperatures. This approach is used to characterize five well‐known conjugated polymers and a small molecule semiconductor, each with distinct thermal phase behavior, where we show good agreement with other thermal characterization methods. Thus, the method we introduce, which we refer to as **A**utomated **T**hermal transition identification using **L**inear regression analysis of the **A**bsorbance **S**pectra (ATLAS), is shown to be an effective and simple screening tool to identify multiple thermal transitions in thin films. This is a high‐throughput thermal analysis approach that will assist in advancing structure‐morphology‐function relations in organic electronics.

## Graphical abstract

This study demonstrates the effectiveness of in situ UV−vis coupled with automated data analysis, namely, ATLAS, for fast and reliable identification of multiple thermal transition in conjugated polymer thin films. This ATLAS method will significantly accelerate the understanding of the phase behavior of D‐A polymers with important implications for structure‐morphology‐property relationships in organic electronics.

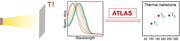

## 1 Introduction

Conjugated polymers and small molecules are the key active materials used in organic electronic devices including organic solar cells (OSCs) [1], organic field effect transistors (OFETs) [2], and organic electrochemical transistors (OECTs) [3]. Innovations in conjugated polymers (CPs) and non‐fullerene molecular acceptors (NFAs) have led to significant improvements in device OSC performance and functionality [4, 5]. An important innovation has been the introduction of electron donating (D) and electron accepting (A) units along the conjugated functional group [1, 6]. This has led to organic semiconductors with complex molecular structures and as a result of complex microstructural ordering and thermal behavior [7]. The thermal and structural characteristics of conjugated materials play a crucial role in dictating the performance and stability of relevant devices [8, 9, 10, 11]. These properties are important for understanding the structure‐morphology‐function relations in organic electronics and improving the thermomechanical and morphological stability of devices [12, 13, 14]. As a result, the molecular packing and structural characteristics of CPs and NFAs have been extensively probed with X‐ray scattering methods [15, 16, 17, 18]. Similar to the in‐depth microstructural analysis, there is a need for an in‐depth understanding of the thermal transitions of these organic semiconductors. This has led to recent efforts to measure thermal transitions in CPs and identify the nature of the observed transitions [14, 19, 20, 21, 22, 23, 24]. This includes efforts to predict the *T_g_* of CPs based on chemical structure using computational methods including machine learning tools [25, 26]. While these approacheFIgus show promise, they often are limited to a subset of CPs and still face challenges in terms of accuracy and broad applicability. What has become evident is that conjugated polymers show complex thermal phase behavior that are often difficult to capture and can often be difficult to be categorized with classic nomenclature [19]. Thus, there remains a need to develop experimental methods capable of screening the large library of D‐A CPs and NFAs and their myriad thermal transitions in a thin film form factor relevant to organic electronics [27].

There are a number of tools that have been used to probe the thermal behavior of organic semiconductors including differential scanning calorimetry (DSC) [28, 29], dynamic mechanical analysis (DMA) [19, 22], spectroscopic ellipsometry (SE) [30, 31, 32], and UV–vis spectroscopy [33]. DSC is a versatile analytical technique to characterize the thermal properties of materials capturing the glass transition (*T_g_*), solid‐liquid crystal (*T_lc_*), cold‐crystallization (*T_cc_*) and melting (*T_m_*) temperatures. However, the amorphous fraction of CPs shows little heat capacity changes upon relaxation making *T_g_* and sub‐*T_g_* transitions difficult to capture with conventional DSC. CPs can also have small degrees of crystallinity or high crystalline disorder making *T_lc_* and *T_m_* difficult to measure [34]. Alternatively, DMA, and in situ SE have been employed with reasonable success in measuring thermal transitions in CPs. Recent demonstrations using DMA have explored the effects of molecular weight (MW) [35], the impact of side chain and backbone modification on relaxation behavior [19], and the role of molecular interactions on blend film behavior [36]. In situ SE has also been used to capture multiple thermal transitions in CPs and small molecules in thin films [20]. Lastly, fast scanning calorimetry (FSC) has recently emerged as a promising tool to determine the thermal signatures of CPs [37, 38, 39]. While these methods can be effective, some tools are not commonly available in research laboratories (FSC, DMA, SE) and can require difficult sample preparation (DMA, FSC) or models to interpret the data (SE) [20, 40].

Absorption spectra of organic molecules provide a wealth of information regarding the chemical structure and molecular organization of the materials being investigated [41, 42, 43], probing both the ordered and amorphous fractions of films. Initial approaches to probe thermal transitions of CPs considered changing absorbance of the films after various thermal treatments [33] to determining the first thermal transition above room temperature [13]. Alternatively, absorbance can be monitored as a function of temperature to observe multiple thermal transitions. This was recently done by Schrickx et al. who measured changes in absorbance of oriented polymer films as a function of temperature and observed numerous thermal transitions in a variety of CPs [44, 45]. In this demonstration, changes in dichroism of the aligned films were used to magnify certain phase changes such as liquid crystal transitions. In‐situ UV–vis experiment was also used to monitor the spectral change of the 0‐0 absorption peak to understand thermally activated twisting in conjugated polymers thin film [46]. These papers highlight some of the strengths of in situ UV–vis spectroscopy. Yet, the demonstrations to date have used manual identification of thermal transitions through visual inspection that leaves ambiguity in the number of thermal transitions and their transition temperature.

In this report, we also probe thermal transitions using UV–vis spectroscopy, but extend the capabilities of the approach by introducing data processing and automated analysis methods that improve the identification of subtle thermal transitions in CPs and NFA small molecules. The approach not only captures multiple transition temperatures but can be used to identify the nature of thermal transitions. The thermal transitions are automatically determined by linear regression analysis of the semilogarithmic representation of the optical deviation metric (DM), along with other spectral changes in absorbance. We refer to this approach as **A**utomated **T**hermal transition identification using **L**inear regression analysis of the **A**bsorbance **S**pectra (ATLAS).

We apply the ATLAS method to analyze six representative systems comprising a largely disordered polymer, two liquid crystalline polymers, two semicrystalline polymers, and an NFA small molecule. Various experimental aspects such as reproducibility, thermal memory, and kinetic effects are investigated. A number of subtle thermal transitions are observed with this approach, yet caution is needed in avoiding false positive transitions near room temperature. We demonstrate that the *T_g_*, *T_lc_*, solid‐solid transition (*T_ss_*), *T_cc_* and *T_m_* are discernible in an automated fashion. The film's absorbance with temperature is complex and fully establishing the physical origin of the changes is beyond the scope of this work. Nevertheless, we show that the change in deviation metric has strong correlation with intra‐ and inter‐molecular optical coupling strengths in two model systems, F8T2 and P3HT. We also find that the change in slope of the linear fits of the semilogarithmic NDM (SL‐NDM) correlates with the type of thermal transition, providing an additional tool to identify various transitions.

Our facile identification of thermal transitions facilitates the creation of a comprehensive data library of intrinsic or processing dependent thermal transitions of conjugated materials. The potential benefits of using in situ UV–vis to probe these transitions are not only speed, reliability and reproducibility but also reduced material usage, and directly probing thin film states rather than bulk materials. Our method demonstrates good sensitivity to probe the thermal characteristics of thin films in either a kinetically quenched state produced from spin‐coating (e.g. different solvents) or from films thermally annealed or solidified from the melt (akin to second heat DSC).

## 2 Deviation Metric and Spectral Character Data Analysis

**FIGURE 1:**
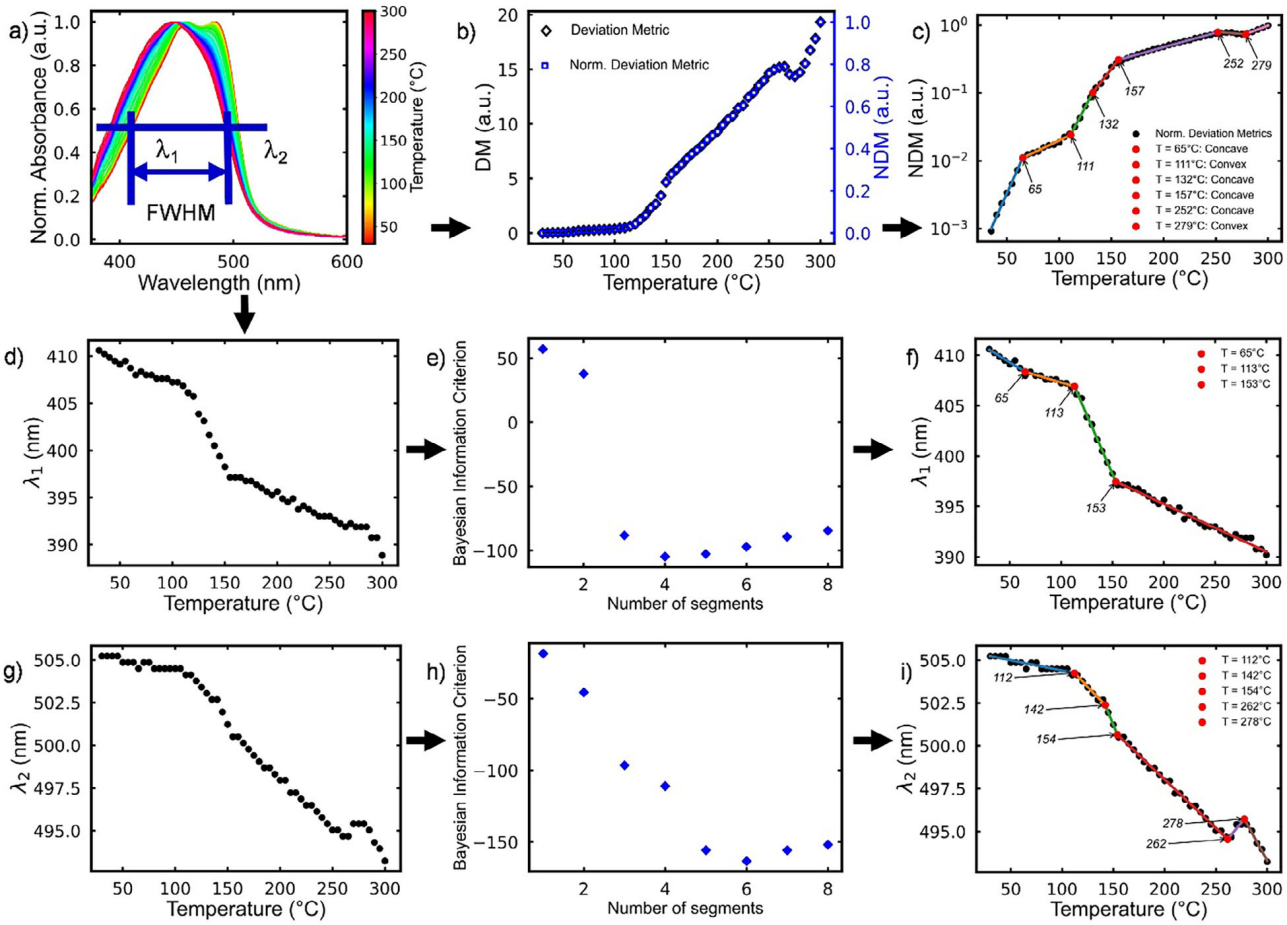
A flowchart describing the steps for automated identification of thermal relaxations and transitions, taking F8T2 as an example. a) Measuring temperature‐dependent UV–vis spectra of thin film with normalized absorbance spectra and a schematic representation of how λ_1_ and λ_2_ are determined. b) Deviation metric (DM) calculated based on Equation (1) along with the normalized deviation metric (NDM) shown on the right y‐axis. c) The NDM with linear segment detection (LSD) that uses piecewise linear regression to determine the linear segments over the temperature range probed. d,g) Temperature‐dependent λ_1_ and λ_2_ extracted from F8T2, respectively; e,h) BIC calculation for λ_1_ and λ_2_, respectively. f,i) Automated determination of thermal transitions using piecewise linear regression for λ_1_ and λ_2_, respectively.

We employ a DM that is defined as the sum of the squared difference between absorbance at a reference temperature and each elevated temperature, given by:

(1) $$DM=\sum_{\lambda_{start}}^{\lambda_{end}}{\left[I_{RT}\left(\lambda \right)-I_{T}\left(\lambda \right)\right]}^{2}\;$$

where λ_*start*_ and λ_*end*_ are the spectral boundaries, *I_RT_* (λ) is the normalized absorbance at the starting temperature that is used as reference temperature (RT), and *I_T_* (λ) is the normalized absorbance at each elevated temperature. This is a modification of a DM first introduced by Root et al. [33] and as given by Schrickx et al. [44] Since the DM considers all the changes in the absorption spectrum as a function of temperature, this parameter captures a broad array of physical changes that have an optical signature such as local molecular packing order and molecular orientation. This results in a highly sensitive tool that captures thermal changes in thin films, albeit without one unique origin as discussed below. A flow chart of the step‐by‐step procedure of the data analysis is shown in Figure 1. The UV–vis absorption spectra of the films were measured over a temperature range of 40°C to 300°C with the normalized spectra shown in Figure 1a. The DM is calculated based on Equation (1) and is then normalized to give a normalized deviation metric (NDM). The DM and NDM exhibit the same shape as representatively shown Figure 1b. However, using NDM brings the advantage of easy comparison across different materials and different heating cycles while having no impact on the thermal transition identification. In order to identifying subtle transitions we analyze the data in a semi‐logarithm (SL) representation. The SL representation is motivated by the fact that many temperature‐dependent processes governing optical coupling, conformational disorder, and thermal expansion in conjugated materials exhibit Arrhenius‐like or exponential behavior. This facilitates the detection of exponential relations (e^aT^), a functional relationship that occurs frequently in nature. When representing the NDM in the SL scale (see Figure 1c), we found that the SL‐NDM data can be represented well by the following expression:

(2) $$\log\left(NDM\right)=C_{n}\;\;+\left\{\begin{array}{c} \;a_{1}\left(T-T_{min}\right),T_{min}<x\leq T_{1}\\ a_{2}\left(T-T_{1}\right),T_{1}<x\leq T_{2}\\ .\\ .\\ .\\ a_{n}\left(T-T_{n-1}\right),T_{n-1}<x\leq T_{max} \end{array}\right.$$

where *n* is the number of linear regions over the temperature range considered. We automatically detect the thermal transitions using PLR [47], a statistical model where the data can be segmented into a series of *n* linear segments (see Figure 1c). The PLR algorithm minimizes the sum‐of‐squares error across all the segments to find the breakpoints [47]. The number of linear segments are determined and the intersections of two linear regression regimes are used to identify a thermal transition. We refer to this fitting as linear segment detection (LSD).

In conjugation with the NDM, spectral changes with temperature were also analyzed. The full width half maximum (FWHM) of the absorbance was determined, and the wavelengths (λ_1_, λ_2_) that defined the FWHM as well as peak absorption wavelength (λ_max_) were analyzed as a function of temperature, with an example given in Figure 1d,g. Specifically, the wavelengths corresponding to half of the maximum normalized absorbance on the shorter wavelength‐ (λ_1_) and higher wavelength‐ (λ_2_) sides are determined. The FWHM is then calculated as the difference between these two wavelengths: FWHM = λ_2_ – λ_1._ The thermal transitions derived from the spectral analysis are also automatically determined using a PLR model. In the spectra analysis, the Bayesian information criterion (BIC) optimization is introduced to automatically determine the optimal number of linear segments. BIC provides a penalty term for the addition of new segments, and we define the optimal number of linear segments to be at the lowest BIC [48, 49]. The equation for BIC is given by:

(3) $$\mathrm{BIC}=n.\ln\left(\frac{SSR}{n}\right)+K.\ln(n)$$

where SSR is the sum of square residuals, n is number of data points, and K is the number of all model‐free parameters that includes 1 slope and 1 intercept for each segment and numbers of breakpoints. Therefore, K is given by:

$$K=2\times \left(\mathrm{segments}\right)+\left(\mathrm{breakpoints}\right)$$

The adoption of BIC enables fully automated transition determinations and reduces human bias from the spectra changes. An example of the automated determination of the thermal transitions from spectra analysis using BIC is shown in Figure 1d‐i. In this case, the minimum BIC value corresponds to the optimal number of linear segments. Similarly, BIC was also applied to the NDM to determine the appropriate segmentation. However, it is important to note that increasing the number of segments consistently leads to a further decrease in both SSR and BIC, therefore, no distinct minimum in BIC was seen in the SL‐NDM plot of some systems (see Figure S2a,b). Consequently, the optimal number of segments can be more effectively determined using the elbow method, wherein additional segments beyond a certain point yield negligible improvements in SSR and BIC. The Python scripts written for PLR analysis of the NDM and spectral features are provided in the Github.

## 3 Results

To evaluate the capability of the ATLAS method to identify the thermal transition of CP thin films, we first present the thermal behavior of PCDTBT, a highly disordered if not amorphous, conjugated polymer [20, 50]. We then evaluate two widely studied liquid crystalline polymers, namely, F8T2 and PBTTT‐C14, and continue with the thermal transitions of two well‐known semicrystalline polymers, namely, P3HT and PNDI2OD‐T2. Finally, we present thermal transitions of an SMA with rich thermotropic properties, EH‐IDTBr. The molecular structures of the materials used are shown in Figure S1.

### 3.1 Disordered Polymer: PCDTBT

**FIGURE 2:**
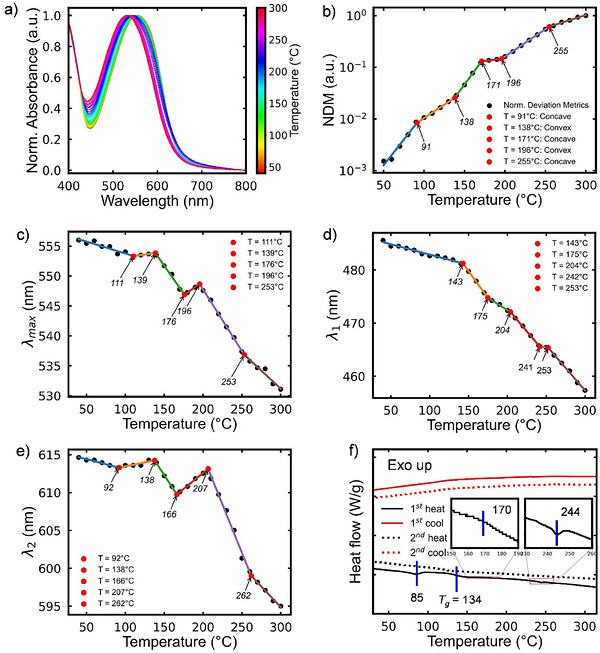
a, b) Temperature‐dependent normalized UV–vis and the corresponding SL‐LSD analysis to detect thermal transitions of the first heat in PCDTBT, respectively. c–e) Temperature‐dependent λ_max_, λ_1_, and λ_2_ extracted from in situ UV–vis spectra, respectively. f) The DSC thermograms of PCDTBT.

The temperature dependent normalized UV–vis spectra of PCDTBT are given in Figure 2a with the absence of vibronic features suggest that PCDTBT is largely amorphous or a disordered polymer [51]. The NDM plot is given in Figure 2b, and spectral changes with temperature are displayed in Figure 2c–e. This data is compared with a DSC thermogram of PCDTBT given in Figure 2f. Five thermal transition temperatures (91°C, 138°C, 171°C, 196°C, and 255°C) are identified from the NDM. The transition observed at ∼138°C is ascribed to the *T_g_* of PCDTBT, in accordance with a the *T_g_* detected at 134°C from DSC as well as the *T_g_* measured at ∼129°C from the peak in the tan δ in the DMA thermogram (Figure S7a). The transition found at 255°C corresponds to the small endothermic peak observed by DSC at 244°C (see inset of Figure 2f). This temperature is likely associated with the clearing temperature of a nematic LC phase based on the work from Gomez et al. who concluded the existence of a LC isotropization transition between 265°C and 275°C [52]. The 138°C and 255°C transitions also match well with spectral shifts observed in λ_max_, λ_1_, and λ_2_ (see Figure 2c–e). The transition at 91°C is in agreement with a broad endotherm peak observed at ∼85°C from DSC. Carefully examining the enlarged region of DSC thermogram shown in the inset of Figure 2f reveals a weak feature at ∼170°C, which is similar to the 171°C transition identified from the SL‐LSD analysis. The transition at 196°C is not detected in the DSC thermogram. However, this transition is in good agreement with a broad tan δ peak found with DMA at ≈180°C (Figure S7a). These additional transitions correlate well with the shifts in λ_max_, λ_1_, λ_2_ as displayed in Figure 2c–e. Overall, transition temperatures observed by other tools (DSC and DMA) are readily observed using the in situ UV–vis automated analysis approach, as summarized in Table S3. In addition, the in situ UV–vis approach is able detect transitions that are not readily discernable by DSC, highlighting the sensitivity of in situ optical spectroscopy in detecting thermal transitions in thin films.

### 3.2 Liquid Crystalline Polymers: F8T2 and PBTTT‐C14

**FIGURE 3:**
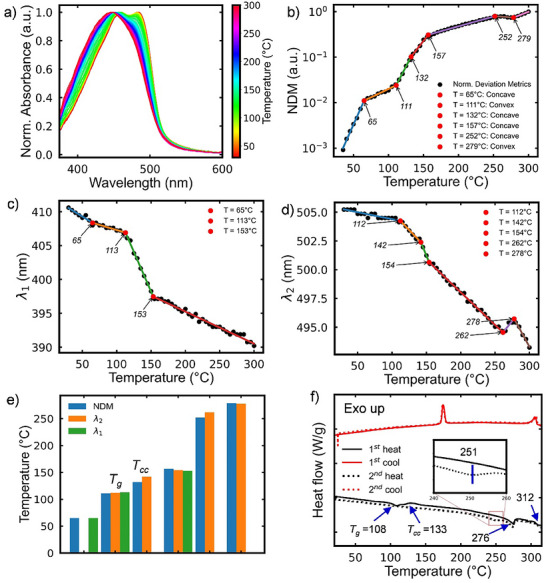
a, b) Temperature‐dependent normalized UV–vis and the corresponding SL‐LSD analysis to detect thermal transitions of the F8T2 film, respectively. c, d) Temperature‐dependent λ_1_, λ_2_ extracted from in situ UV–vis spectra, respectively. e) Comparisons of thermal transition determined from NDM, λ_2_ and λ_1_ analysis. f) The DSC thermograms of F8T2.

We consider the polymers F8T2 and PBTTT‐C14, both of which have been shown to have rich thermotropic LC characteristics [44, 53]. The SL‐LSD of the NDM for F8T2 is given in Figure 3a,b. The SL‐LSD analysis indicates six thermal transitions. To assist in defining the transitions, we consider the spectral changes in absorbance features. The temperature‐dependent λ_1_ and λ_2_ plots are shown in Figure 3c,d, respectively. All transitions defined by the SL‐LSD analysis of the NDM have complimentary transitions in λ_2_, as displayed in Figure 3e, except for the transition at 65°C, which is only observed in the NDM and λ_1_. The low temperature transition at 65°C is confirmed by DMA at around 64°C. The λ_1_ is in the shorter wavelength part of spectra, a region typically associated with more disordered component of the film, as demonstrated in P3HT [54]. Thus, the change in λ_1_ suggests a transition related to disordered segments. We further summarize all thermal transitions observed by UV–vis, DSC, and DMA in Table S4. The 111°C transition is ascribed to the *T_g_* of F8T2, in agreement with DSC and DMA. The 132°C transition is assigned to the *T_cc_* of F8T2, consistent with an exothermic transition observed by DSC and an increase in storage modulus at a similar temperature observed in the DMA (Figure S8a). The transition at 279°C is consistent with the endotherm at 276°C from DSC in both 1st and 2nd heat cycles, which was previously assigned to a transition into an LC [55]. The transition at 252°C in the NDM is similar to an endotherm peak at 251°C observed in DSC. A clear transition is observed from the SL‐LSD and spectral analysis at approximately 157°C, which does not have a corresponding transition in DSC or DMA, highlighting the sensitivity of the measurement. There is a distinct change in tan δ in DMA near 150°C, which is possibly related but requires further analysis. An endotherm at 312°C assigned to the LC isotropization is observed in the DSC thermogram but beyond the current limit of our UV−vis testing stage.

**FIGURE 4:**
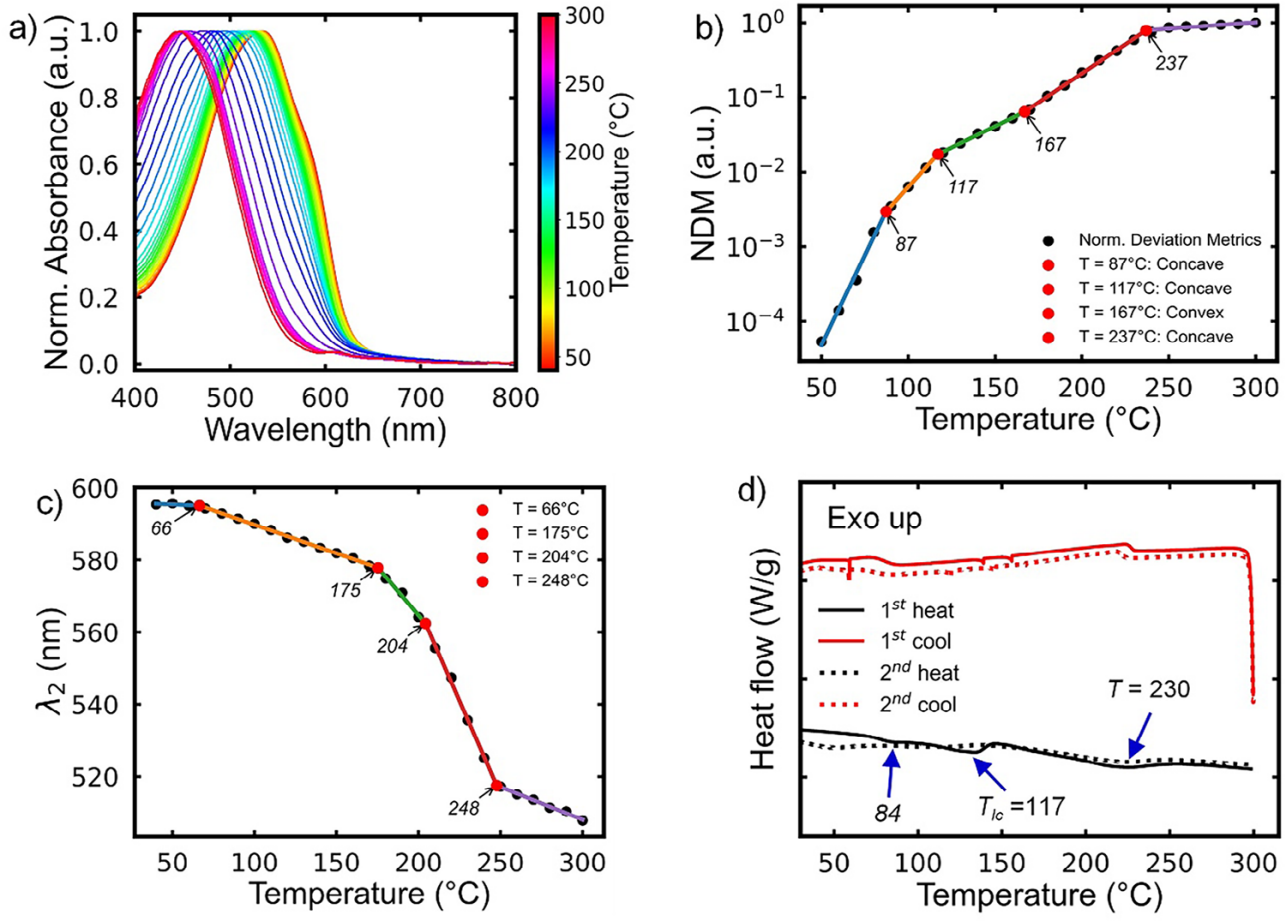
a, b) Temperature‐dependent normalized UV–vis and the SL‐LSD analysis to detect thermal transitions of the first heat in PBTTT‐C14 film, respectively. c) Temperature‐dependent λ_2_ extracted from in situ UV–vis spectra. d) The DSC thermograms of PBTTT‐C14.

The UV–vis, SL‐LSD analysis, temperature‐dependent λ_2_ and complementary DSC thermogram of PBTTT‐C14 are shown in Figure 4a–d, respectively. Here, the SL‐LSD analysis method identifies four thermal transition temperatures at 87°C, 117°C, 167°C, and 237°C. Similarly, λ_2_ identifies four transitions at 66°C, 175°C, 204°C, and 248°C. The 237°C SL‐LSD transition is assigned to the order–disorder transition of the liquid, matching with the broad endothermic peak at around 230°C shown in DSC and at a similar temperature observed in the DMA (Figure S9a), consistent with other reports [53, 56]. This transition is consistent with changes in λ_1_, λ_2_, λ_max_ and FWHM detected at around 243°C–248°C (see Figure 4d; Figure S9b–d). We note when the absorption edge λ_2_ approaches the spectrometer limit at elevated temperatures, FWHM values are only reported when both half‐maximum points are fully captured within the measured spectral window. A broad endothermic peak in the DSC thermogram at ∼84°C is observed in the 1st heat cycle that shifts to ∼50°C in the 2nd heat, matching the transition at 87°C found in the SL‐LSD analysis. The 117°C transition is assigned to the liquid crystal transition (*T_lc_*), matching another endotherm peak at ∼120°C in the DSC thermogram. The 167 °C is noted from SL‐LSD and also observed in the spectral shift (λ_2_ at 175°C) but absent from DSC analysis. Yet, it is similar to the broad thermal relaxation found by DMA at ∼ 160°C. A similar transition has also been previously observed by FTIR, which was attributed to a LC‐LC phase transition [57]. The 117°C and 167°C transitions from SL‐LSD analysis occur at temperatures where prior in situ GIWAXS studies reported changes in lamellar spacing [58]. Again, we find consistency between the thermal transitions probed between our in situ UV–vis method and the various characterization methods reported in prior works, as summarized in Table S5. We note that ATLAS does not directly resolve crystalline or liquid crystalline structures; rather, it identifies thermal transition temperatures, which may be interpreted by comparison to complementary structural characterization such as GIWAXS reported in prior studies.

### 3.3 Semicrystalline Polymers: P3HT and PNDI2OD‐T2

**FIGURE 5:**
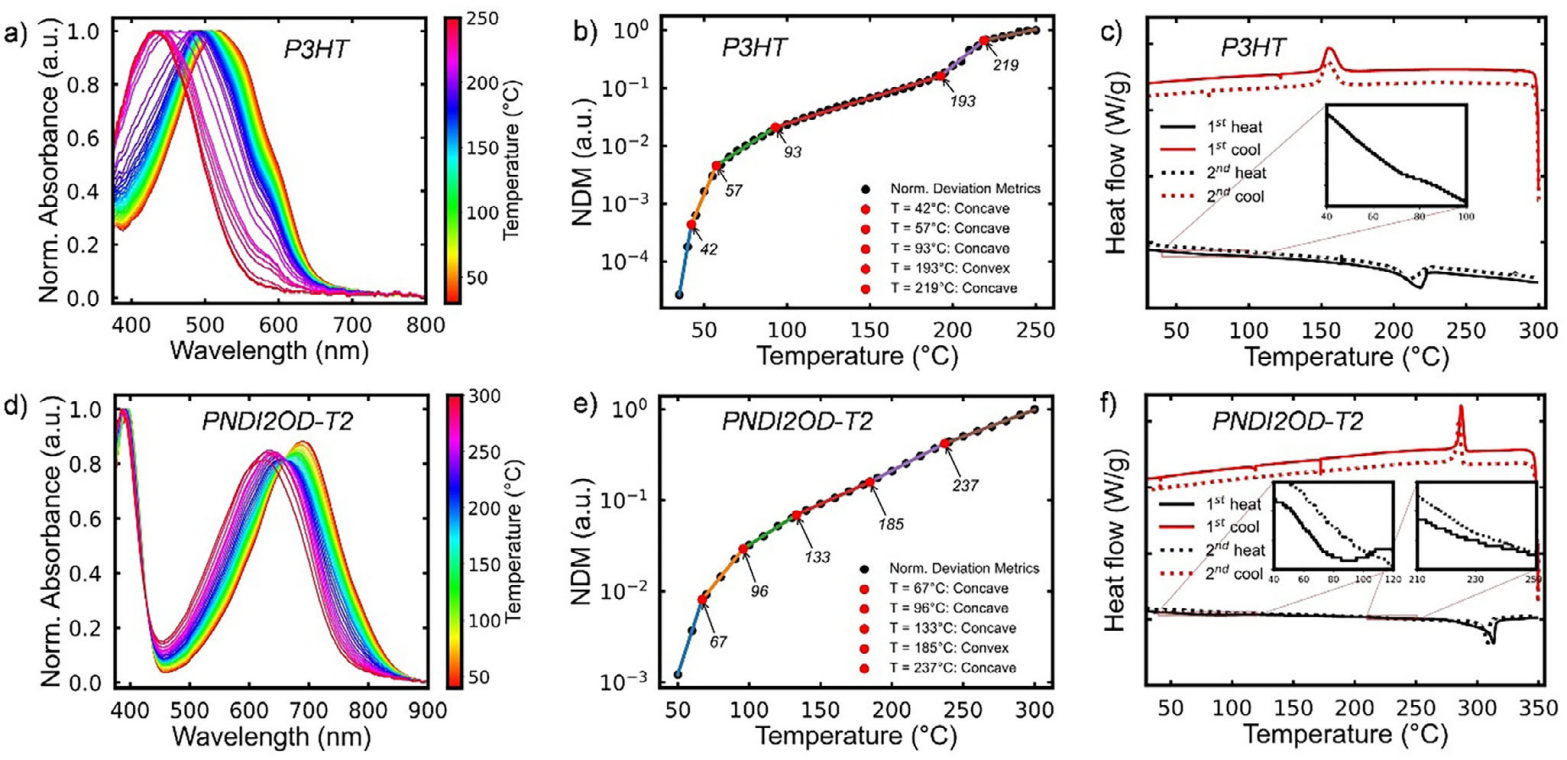
a, d and b, e) Temperature‐dependent normalized UV–vis and the corresponding SL‐LSD analysis to detect thermal transitions of the first heat in P3HT and PNDI2OD‐T2 films, respectively. c,f) The DSC thermograms of P3HT and PNDI2OD‐T2, respectively.

We consider two well‐known semicrystalline polymers, namely P3HT and PNDI2OD‐T2. The normalized UV–vis of P3HT and corresponding NDM are shown in Figure 5a,b. Several transitions are detected from SL‐LSD analysis, in contrast to only a single endothermic peak observed in the DSC thermogram. Several thermal transitions in P3HT have been identified using different approaches as summarized in Table S6. The transition at 220°C is ascribed to *T_m_* in accordance with endotherm peak at 220°C from DSC (Figure 5c) and the tan δ peak found at ∼220°C from DMA (Figure S10a). The 193°C transition is consistent with change in thickness at 196°C monitored with in situ SE [20]. This transition could be related to the *T_g_* of the rigid amorphous fraction, based on the previous work from Martin et al. where the authors estimated the *T_g_* of the rigid amorphous fraction in P3HT to be ∼180°C by taking advantage of the enthalpy recovery process from FSC [39]. Two low temperature transitions detected at 47°C and 62°C could be correlated with broad endotherm from DSC trace (see inset in Figure 5c). The 62°C and 101°C are consistent with the changes in the thickness monitored with in situ SE [20, 59], with a slight discrepancy likely due to difference in measurement protocol.

The in situ normalized UV–vis spectra and corresponding NDM plot of PNDI2OD‐T2 are shown in Figure 5d,e. The SL‐LSD analysis identifies five transition temperatures. In contrast, only a single prominent endotherm peak corresponding to *T_m_* at ∼310°C is observed in the DSC thermogram (Figure 5f). The transition identified at 237°C is similar to the solid‐solid transition temperature (*T_ss_*) previously observed at 232°C using in situ GIWAXS [60], as well as endothermic‐like slope changes in the DSC trace (see inset Figure 5f). The 67°C transition observed in the UV–vis measurement match with the tan δ peak found at ∼65 °C from DMA thermogram (Figure S11a). The 67°C and 96°C from NDM could be related to the broad endotherm peak ranging from 60°C to 110°C in DSC (see inset Figure 5f). Two other transitions at approximately 133°C and 183°C cannot be confidently assigned to specific transition at this time, but coincide with thermal expansion coefficient changes observed by in situ SE that occur at 120°C and 180°C [61]. The spectral analysis of PNDI2OD‐T2 reveals changes in λ_1_ and λ_2_ at ∼180°C and ∼280°C (Figure S12a,b).

We acknowledge that some lower‐temperature thermal transitions estimated from SL‐NDM associated with one or more consecutive concave transitions in semicrystalline P3HT and PNDI2OD‐T2, are less distinct, indicating the possibility of a false positive or over‐interpretation in our SL‐LSD results. Caution is suggested in identifying thermal transitions near room temperature with this method. Decreasing the temperature steps and starting from lower temperatures might minimize the risk of false positives and support the presence of unique transitions that other methods might miss. Nevertheless, the transition temperatures in P3HT and PNDI2OD‐T2 determined by our ATLAS method are very consistent with those reported in the literature, as summarized in Tables S6 and S7.

### 3.4 Small Molecule Acceptor: EH‐IDTBr

**FIGURE 6:**
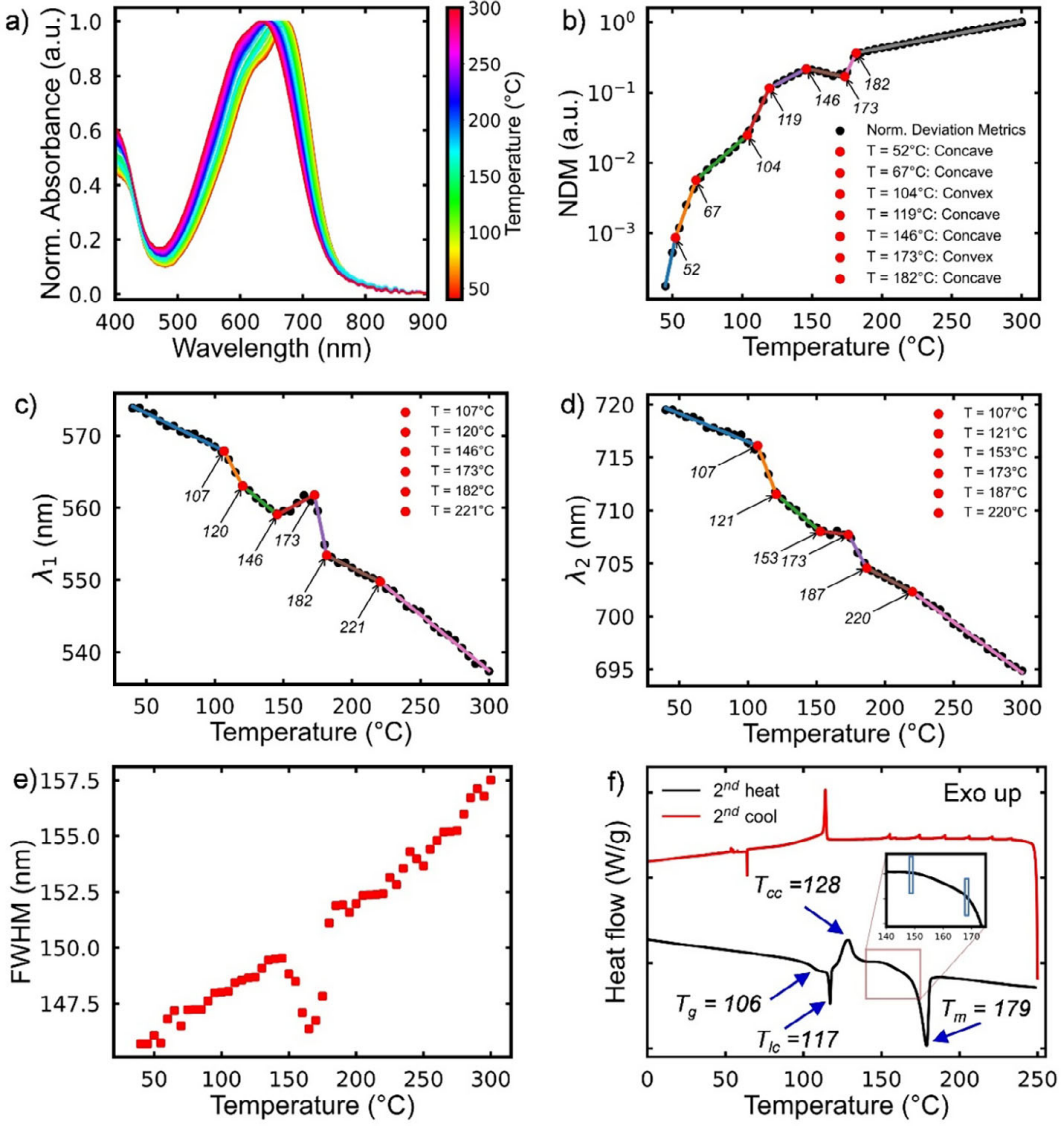
a,b) Temperature‐dependent normalized UV–vis and the corresponding SL‐LSD analysis to detect thermal transitions of EH‐IDTBr film, respectively. c–e) Temperature‐dependent λ_1_, λ_2_ and FWHM extracted from in situ UV–vis spectra, respectively. f) The DSC thermograms of EH‐IDTBr.

We extend the analysis to non‐polymeric semiconductors by considering EH‐IDTBr, a NFA that displays rich thermotropic properties in DSC [20]. The temperature‐dependent UV–vis spectra of EH‐IDTBr with the NDM are displayed in Figure 6a,b. Seven thermal events are identified by the SL‐LSD analysis. In comparison, the second heat DSC thermogram shows four distinct, obvious thermal transitions or events: a *T_g_* at ∼106°C followed by *T_lc_* at ∼117°C, a cold‐crystallization (*T_cc_*) at ∼128°C and *T_m_* at ∼179°C, as shown in Figure 6f. We note that the 1st heat DSC from sample does exhibit similar thermal transitions to the 2nd heat, with very sharp and clear peaks found in the 2nd heat after solidification from the melt (see Figure S13). The comparison in thermal transitions determined from UV–vis analysis and DSC is summarized in Table S8. It is clear that the transitions observed at 104°C and 119°C are the *T_g_* and *T_lc_*. A transition is also observed at 182°C, which matches well with endothermic transition, *T_m_* found in the DSC thermogram. These three transitions are also apparent when considering the spectral shifts λ_1_ and λ_2_ as function of temperature, as shown in Figure 6c,d. Two other transitions at 146°C and 173°C are noted from SL‐LSD analysis, which are consistent to temperatures where shifts in λ_1_ occur at 146°C and 173°C, respectively, and shifts in λ_2_ occur at 153°C and 173°C, respectively (Figure 6c,d). Carefully examining the enlarged region of DSC thermogram shown in inset Figure 6f reveals two subtle exothermic‐like slope changes at ∼150°C and ∼170°C, which seem to match well with transitions detected from in situ UV–vis. Another low temperature relaxation may be assigned at temperature range of 52°C–67°C, but is difficult to conclude.

### 3.5 Considerations of Experimental Conditions

**FIGURE 7:**
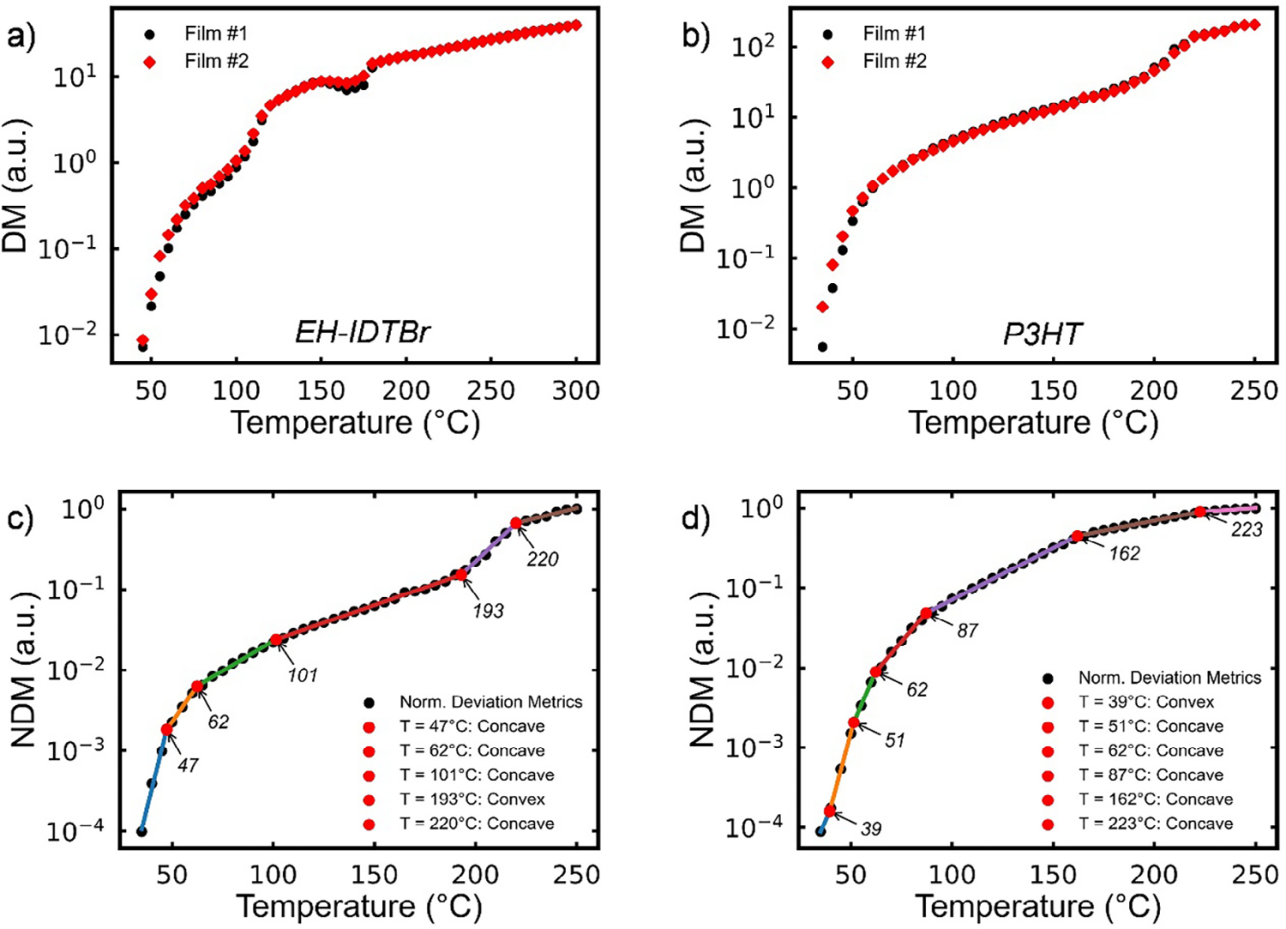
a, b) Deviation metrics of two independent films in EH‐IDTBr and P3HT thin films, respectively. c, d) SL‐LSD analysis to detect thermal transitions of the 1st and 2nd heat cycle in P3HT film, respectively.

To explore the reproducibility, reliability, and heating rate sensitivity of our ATLAS method in measuring thermal transition of conjugated materials thin films, we varied experimental parameters in some representative systems. Regarding reproducibility, duplicate independent experiments and SL‐LSD analysis on EH‐IDTBr and P3HT films are provided in Figure 7, Figure S14, and Table S9. There is good agreement between the identified transition temperature for the separate measurements.

Since the thermal memory of bulk polymers often has an impact on thermal transition temperatures, for instance, *T_m_* or *T_g_*. We compared the thermal transitions from the NDM between the 1st and 2nd heat cycles in P3HT and PNDI2OD‐T2, displayed in Figure 7 and Figure S15, respectively. In both cases, there are clear changes in the shape of the NDM, yet there remain common transition temperatures. In P3HT, there is a similar *T_m_* detected between the 1st heat (220°C) and 2nd heat (223°C), which is consistent with the DSC analysis (see Figure 5c). There is also consistency in detected transition temperatures at low temperature regimes between the two cycles (Figures S15 and S16). For PNDI2OD‐T2, we find that the solid–solid transition in the 1st heat is consistently observed in range of 230°C–240°C. The *T_ss_* shifts as expected given the different polymorphs present in the film in the 2nd heat. The lower temperature transitions remain largely consistent between the two heat cycles. The slight variation in transition temperatures between the two cycles in P3HT and PNDI2OD‐T2 suggests good consistency in the detection method, despite the thermal history being erased.

We also investigated how measurement parameters such as scan‐rate influence NDM determined thermal transition temperatures [62, 63, 64]. In conventional DSC, transitions will generally shift to a higher temperature when heated at a higher rate because of the kinetic nature of these transition events [64, 65]. The shift of the peak to higher temperatures is known as thermal lag. We explored the kinetic behavior on PNDI2OD‐T2 using in situ UV–vis with three different heating rates (T‐ramp) of ∼5, ∼10, and ∼15°C/min (see Figure S17). Five thermal transitions are clearly discernible from the SL‐NDM analysis with T‐ramp of 5 and 10°C/min, whereas only four transitions are detected from SL‐NDM with T‐ramp 15°C/min. The ∼180°C transition is no longer identified at high scan rates. This transition was weak at the lower scan rate, and likely smears out over a larger temperature range at high heating rate, reducing the ability to identify the transition. We also introduced an additional isothermal step at each temperature to ensure thin film reaches a thermal equilibrium state in PNDI2OD‐T2 films. Two thin film sets, one with continuous heating (no isotherm step) from 40°C to 300°C and another with the same heating step followed by a 2‐min isothermal step, were compared, both with the same overall heating rate. The analysis with the transition temperatures is shown in Figure S18. These experiments exhibit a high level of similarity in the probed transitions, with five transitions observed. This demonstrates that additional isotherm treatment is not necessarily required for the measurement method.

## 4 Discussion

### 4.1 Physical Origin of the Temperature Dependent Absorbance

There are many possible contributions that lead to changes in the absorbance signature of the organic semiconductor films. An exhaustive analysis is beyond the scope of this work, however, here we consider how the changes in absorption character with temperature is related to intra‐ and inter‐molecular electronic coupling strength in these materials [54, 66]. We consider two polymers, F8T2 and P3HT as two model systems that have clear vibronic features that are known to be associated with molecular ordering [67, 68].

**FIGURE 8:**
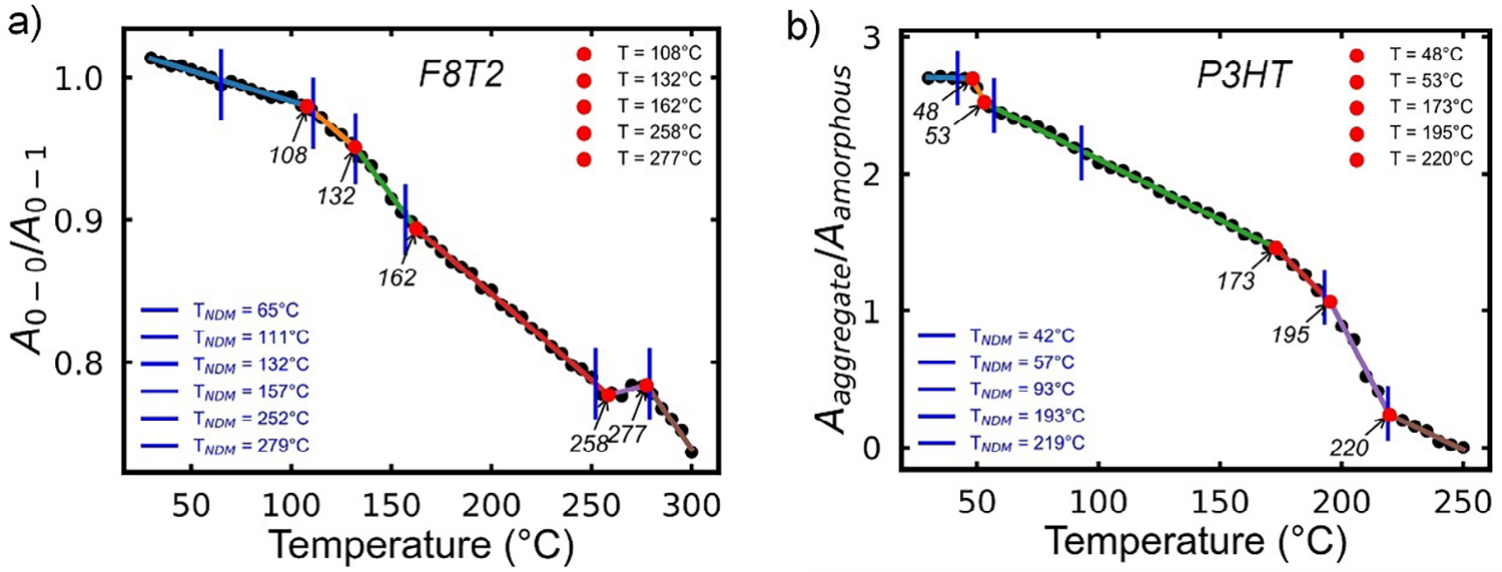
a) PLR analysis on the absorbance ratio of two vibronic peaks A_0‐0_/A_0‐1_ in F8T2 polymer film. b) PLR analysis on the absorbance ratio of between aggregates and amorphous fractions in P3HT polymer film. (The blue line indicates the transition temperatures determined from the SL‐NDM analysis).

The UV–vis spectra of an F8T2 thin film shows two clear vibronic peaks (Figure 4a). Based on Spano et al.’s model, the strength of inter‐chromophore coupling can be extracted from the relative height of the vibrational peaks in the absorption spectra [54, 66]. For simplicity, the wavelengths associated with the 0–0 and 0–1 transitions are estimated at 30°C and assumed to remain unchanged with temperatures. The relative strength ratio of 0–0 and 0–1 vibronic peak of F8T2 polymer were calculated and shown in Figure 8a. As the temperature increases, it is observed that the 0‐0 vibronic feature gradually vanishes, reflecting a change in the oscillator strength of the aggregated species. Interestingly, there are distinct changes in A_0‐0_/A_0‐1_ that are well correlated with the thermal transitions identified in the SL‐NDM analysis, see Figure 8a. In the case of P3HT, we identified the fraction of the film absorbance associated with aggregates and amorphous portions of the film using a previously described process [69], and outlined further in Section S2 of the SI. We observed the changing trend in absorbance ratio of between aggregates and amorphous fractions (A_aggregate_/A_amorphous_) is reversely proportional to the change in the NDM. The automated PLR analysis is performed on A_aggregate_/A_amorphous_ and similar thermal transitions are identified to the SL‐NDM analysis, see Figure 8b.

The detailed analysis on F8T2 and P3HT suggest the DM is strongly related to change in intra‐ and inter‐molecular electronic coupling strength, and that across a thermal transition there is a distinct temperature dependence of the electronic coupling, possibly through changes in aggregate ordering and/or thermal expansion. The optical signature of these materials holds a wealth of information, and continued exploration of this behavior may uncover more detailed structural changes associated with the observed thermal transitions.

### 4.2 Empirical Signature of Thermal Transitions Determined by In Situ UV–Vis

Assigning the nature of thermal transitions in DSC is well established by analyzing the changes in the heating required to change the temperature of the material, including latent endothermic and exothermic transitions, and through comparing heating and cooling cycles, and first and second heat cycles. In some cases, modulation methods or complex aging protocols are used to improve sensitivity or ability to assign a transition. Modulation and scan rate dependence like those used in DSC should also be beneficial to make assignments based on UV–vis data alone. We are at the very beginning of understanding the power of UV–vis analysis and full understanding and utilization of the method will likely take time. Yet, an interesting and potentially powerful tool might be identifying the nature of a transition temperature by examining how two linear segments intersect. The intersection can be simply described as convex (increasing slope with increased temperature) or concave, and we generally observe the NDM signature will be opposite to the spectra shifts given the nature of the analysis. One average, the spectral shifts are to smaller wavelength, i.e. negative, whereas the NDM is defined as a positive parameter. If the spectral shifts were shown as energy changes, the general trends would be the same, and most spectral changes would be concave. To establish if these changes are meaningful, we compare the convex or concave characteristics with known transitions using DSC as a reference tool, and by focusing in part on the less frequent transitions that are convex in the NDM.

#### 4.2.1 Melting or Clearing Transition (T_m_)

*T_m_* is a thermodynamic quantity generally reported as a single value, estimated at the end‐point of the endothermic peak in the DSC thermogram. Using the SL‐LSD analysis, *T_m_* estimated at the point where a change in slope is detected in the NDM and/or the spectral features. It likely represents the effective sample average and not the onset or endpoint of melting. All measured *T_m_* exhibit a drop in linear segment slope in the NDM (slope approaches zero), often accompanied by a plateau region above the melting or clearing temperatures. As the conjugated materials enter the isotropic state, their effective conjugation length might remain unchanged with further temperature increases, resulting in minimal changes in the UV–vis spectra beyond *T_m_*. This is most clearly seen in PBTTT‐C14. In contrast, PCDTBT, P3HT and EH‐IDTBr exhibit a linear region of SL‐NDM with small slopes above the melting or clearing transition. There might be a range of slopes above *T_m_* that could be considered as an indicator to recognize the melting or clearing transition, but further studies are needed. The slope is likely related to thermal expansion in the melt or how easily thermal energy can further reduce the intramolecular coherence length of the molecules. In addition, *T_m_* is likely the last concave shape in the probed temperatures ranges, similar to the last endotherm peak found in DSC thermogram. Similarly, the *T_m_* can be often associated with the convex change in the spectral characteristics.

#### 4.2.2 Solid‐Liquid Crystalline Transition (T_lc_)

We observed a similar signature to that observed in melting or clearing for the solid‐liquid crystalline transitions, *T_lc_*, with concave shape in SL‐NDM. This observation seems reasonable since both *T_m_* and *T_lc_* are exothermic transitions from DSC analysis and considered first‐order transitions. The difference to the *T_m_* transition is that the slopes of the linear segments are larger for *T_lc_*. Data from a larger survey of materials might indicate if a specific range of slopes of the two abutting segments or the change in slopes can be used to make an assignment unambiguously and intrinsically to the UV–vis data.

#### 4.2.3 Glass Transitions (T_g_)

Glass transitions involve subtle baseline changes in the heat flow in DSC thermogram. Sample heterogeneity can result in a gradual or multi‐step baseline shift instead of a sharp step change; it is therefore challenging to exactly pinpoint the glass transition. *T_g_* is often estimated as the midpoint of two tangent lines in the heat flow before and after the transition. In contrast, the broadening is not observed in the SL‐LSD method allowing to pinpoint an effective *T_g_* as a single value at the intercept where there is a significant change in slope. In contrast to *T_m_* and *T_lc_*, we found that *T_g_* is generally associated with a convex shape in the SL‐LSD analysis. Examples are PCDTBT at 138°C, F8T2 at 111°C, and EH‐IDTBr at 104°C, even though subtle concave at 38°C in 2nd heat of P3HT. Until an exception is found, this empirical assignment rule might be used to identify the *T_g_*.

#### 4.2.4 Low Temperature Transitions

Some lower‐temperature thermal transitions in NDM associated by one or more concave transitions, are less distinct. Rather than two distinct transitions, there might one thermal transition that may represent a single broad transition. This broadening likely reflects the nature of the transition, which lacks a sharp change and, therefore, complicates precise identification, potentially leading to error or misinterpretation in our SL‐LSD results at low temperature regime in some cases. For example, in the EH‐IDTBr film, two continuous concave shapes observed in the SL‐LSD thermogram at 52°C and 67°C might not truly represent two separate transitions. Instead, the midpoint of this range (∼60°C) could represent a single transition. The same scenario may be applied to P3HT and PNDI2OD‐T2 where the midpoint of two of the lowest transitions could be a single transition. Therefore, additional analysis on the deconvolution of UV–vis spectra (e.g., peak decomposition, peak positions) together with complementary characterization are required to confirm the existence of SL‐LSD‐derived low thermal transition temperatures. Overall, we note that these low temperature transitions are universally observed in the metrics analyzed. Transitions in the 40°C to 70°C range are likely related to sidechain melting/relaxation or physical ageing of glassy material regions, as observed with the endotherm peak in DSC or tan δ peak in DMA [19, 35, 56]. In the case of FTAZ, it has been previously shown that a very weak endothermic peak is observed at 50°C, at which point unique scattering signal associated with the sub‐lattice crystallization disappears in the GIWAXS diffraction pattern [70]. It was also shown by NEXAFS and GIWAXS that such side‐chain crystallization occurs in P3HT, PNDI2OD‐T2, and PCDTBT in drop‐cast samples [70]. The sidechain ordering seems depend on the nature of sidechain configurations with branched sidechains are less likely crystallize than the linear sidechain. If the side‐chain crystallization is pervasive, the UV–vis and NEXAFS spectra change completely due to competing ordering between the backbone and the sidechains at low temperatures. We however note that the NDM and spectral changes are very small for these transitions, and we surmise that only a small fraction of materials have crystallized side chains that melt in the 40°C–70°C temperature range when materials are cast into a film.

### 4.3 Unique Advantages of In Situ UV–Vis: High‐Throughput Characterization with Automated Data Analysis

Thermal transitions probed by our in situ UV–vis methodology are parallelly compared to DSC, FSC, DMA, and in situ SE. The automated method uncovers numerous transitions that were not discernable by conventional DSC, but were verified by other tools including in situ SE [20, 61]. Thus, this approach is a powerful tool to capture the complex thermal behavior of conjugated polymers and small molecules. One caution is that in some cases, the low‐temperature thermal transitions estimated from the NDM may be a false positive or over‐fitting of the data. Thus, transitions near room temperature require careful consideration, which may include comparison to other complementary techniques. For reliable ATLAS measurements, films should be semi‐transparent (absorbance ≤ 1–1.5), uniform, and sufficiently thin (typically <200 nm) to minimize optical saturation and thermal lag. Thin glass substrates and moderate heating rates ensure thermal equilibration at measured spot.

Nevertheless, the in situ UV–vis has a number of unique advantages beyond sensitivity including the ability to probe thermal transitions directly from device‐relevant thin films, which is challenging with DMA or DSC. We note that polymer dispersity may broaden or shift thermal transitions, however, ATLAS captures the effective thin‐film response relevant to device processing, where such dispersity is unavoidable. The ATLAS approach can therefore be used in a similar fashion to FSC and in situ SE to probe thermal transitions in thin films but with faster data acquisition and simpler and more direct data interpretation using automated data analysis. The thin film characterization provides a facile route to investigate the confinement effect not only on *T_g_* but also to extended to *T_lc_* or *T_ss_* in conjugated materials which is a challenge with the existing tools [71]. The measurement requires modest equipment and can be readily implementable in most laboratories. The approach is compatible with screening many films relatively quickly, possibly through robotic experimentation methods [72, 73], which would couple well with the automated data analysis, resulting in a high‐throughput experimentation tool. Though in situ UV–vis offers significant advantages over existing techniques, it currently cannot probe thermal transitions below room temperature. Future improvements by using a cryo‐stage should enable this capability. Lastly, further analysis of the changing absorbance spectra may be a route to capture the origin of the relaxations/transitions of conjugated materials.

## 5 Conclusion

We have demonstrated that in situ UV–vis with automated data analysis is not only capable of replicating information observed in DSC analysis but can also obtain information on structural changes that do not produce a significant heat flow change to be discernable in conventional DSC. This in situ UV–vis method, we refer to as ATLAS, has shown beneficial characteristics such as speed, reliability, and reproducibility in measuring a range of thermal behaviors in largely disordered, liquid crystalline, and semicrystalline CPs, as well as thin films of conjugated small molecules. We have developed and validated our protocol with different experimental conditions to acquire highly reproducible characterization of thermal transitions. In short, ATLAS is a facile and robust tool in determining thermal transitions of molecular thin films. Importantly, the ATLAS method interrogates the materials in a thin film form factor used in devices, a task considerably more challenging with DSC or DMA. A key advantage is thus the fast determination of thermal transitions of the kinetically quenched states produced via coating techniques, knowledge which can then be used to optimize the device characteristics, for example, maximizing the charge transport and optoelectronic properties via post‐processing treatment such as thermal annealing. Better understanding the thermal characteristics of conjugated materials in thin films might also prove impactful for predicting general structure‐processing‐property relations in electronic devices. Its compatibility with high‐throughput characterization will allow for the ready creation of a comprehensive data library on thermal transition of conjugated materials, a library that should be extremely useful for effective training and using ML/AI structure‐properties‐function models. The large dataset could be used, for example, as input data and descriptors that help establish molecular factors that drive various thermal transitions. Furthermore, applying ML algorithms in analyzing the large dataset should enable the prediction of thermal transition of conjugated materials as a function of chemical structure, speeding up materials discovery for organic electronics.

## 6 Experimental Section

The synthesis procedure of polymer PNDI2OD‐T2 and its detailed structural characterization was fully described in prior work [74]. The P3HT and F8T2 were acquired from Ossila. PBTTT‐C14, PCDTBT, and EH‐IDTBr were acquired from 1‐Material. Further details such as molecular weight and lot numbers are provided in the Supporting Information. All materials were used without further purification. Glass substrates were cleaned via ultrasonication in deionized water, acetone, and isopropanol for 10 min, respectively, and then cleaned with UV‐ozone for another 20 min. Polymers PCDTBT, F8T2, PBTTT‐C14, P3HT, PNDI2OD‐T2 were dissolved in chlorobenzene at concentration of 15 mg/mL. EH‐IDTBr was dissolved in chlorobenzene at concentration of 20 mg/mL. All solutions were heated at 70°C for 4h before deposition. All films were cast onto UV–ozone‐treated glass substrates at 1500 rpm for 60 s, followed by drying under nitrogen environment prior to thermal measurements. Unless otherwise noted, films were measured in the as‐cast state. The UV–vis absorbance of the film was recorded using an Ocean Optics Jazz spectrometer. The film was held under vacuum and heated using a Linkam heating stage. Absorbance was measured at a heating rate of ≈10°C/min in most experiments unless otherwise stated. Films were mounted directly on the Linkam heating stage under vacuum without conductive oil or paste. Temperature calibration using a contact K‐type thermocouple confirmed excellent agreement between the film surface temperature and the nominal stage temperature (see Section S5 for details). Vacuum was used to avoid possible effects of photooxidative degradation at elevated temperatures. The temperature range was limited to 300°C, which was not high enough to reach the melt of some materials. The DSC thermograms were collected with a TA Instruments Discovery DSC. The heating/cooling rate was 10°C/min with N_2_ flow. Baseline and temperature were calibrated with sapphire and indium. DMA was performed with a TA Instruments DMA 850. The 15 mg/mL solution of polymers was drop‐cast onto a glass mesh with experimental details outlined elsewhere [19, 36]. All the temperature scans were run at an oscillation frequency of 1 Hz, an oscillation strain of 0.1%, and a temperature ramp of 3°C min^−1^. Thermal transition temperatures were identified from the tan δ curve.

## Conflicts of Interest

The authors declare no conflicts of interest.

## Supporting information




**Supporting File**: smtd70612‐sup‐0001‐SuppMat.docx.

## Data Availability

The data that support the findings of this study are available in the supplementary material of this article. The Python scripts used for ATLAS analysis are available at: https://github.com/DoanVu20/ATLAS‐Automated‐Thermal‐Transition‐Identification.
