# Supplementary material for: Automated Identification of Thermal Transitions in Conjugated Material Thin Films Using In Situ Optical Spectroscopy
Source: Small Methods. 2026 Mar 31;10(14):e02209. doi: 10.1002/smtd.202502209 (PMC13397149; doi:10.1002/smtd.202502209)
Supplement: Supplementary file 1 — Supporting File: smtd70612‐sup‐0001‐SuppMat.docx. [file SMTD-10-e02209-s001.docx]

Supporting Information

Automated Identification of Thermal Transitions in Conjugated Material Thin Films using In-situ Optical Spectroscopy

*Doan Vu^1,2^, Martyn Jevric,^3^ Alireza Samadani,^2^* *Mats R. Andersson,^3^ Christopher R. McNeill,^4^ Brendan T. O’Connor ^2^*^*^*, and Harald Ade^1^*^*^

D. Vu, Prof. H. Ade

Department of Physics and Organic and Carbon Electronics Laboratories (ORaCEL), North Carolina State University, Raleigh, NC 27695, USA

Email: [hwade@ncsu.edu](mailto:hwade@ncsu.edu)

D. Vu, A. Samadani, Prof. B. T. O’Connor

Department of Mechanical and Aerospace Engineering and ORaCEL, North Carolina State University, Raleigh, NC 27695, USA

Email: [btoconno@ncsu.edu](mailto:btoconno@ncsu.edu)

M. Jevric, Prof. Mats R. Andersson

Flinders Institute for Nanoscale Science and Technology, Flinders University, Adelaide, South Australia 5042, Australia

Prof. C. R. McNeill

Department of Materials Science and Engineering, Monash University, Clayton, Victoria 3800, Australia

**S1. Materials**

All polymers are widely studied benchmark materials with well-established chemical structures reported in the literature.

**Abbreviations**

- PCDTBT: *Poly[N-9′-heptadecanyl-2,7-carbazole-alt-5,5-(4′,7′-di-2-thienyl-2′,1′,3′-benzothiadiazole)*
- F8T2: *Poly(9,9-dioctylfluorene-alt-bithiophene)*
- PBTTT-C14: *Poly(2,5-bis(3-tetradecylthiophen-2-yl)thieno[3,2-b]thiophene)*
- P3HT: *Regioregular poly(3-hexylthiophene-2,5-diyl)*
- PNDI2OD-T2: *Poly{[N,N'-bis(2-octyldodecyl)naphthalene-1,4,5,8-bis(dicarboximide)-2,6-diyl]-alt-5,5'-(2,2'-bithiophene)}*
- EH-IDTBr: *(5Z)-3-ethyl-2-sulfanylidene-5-[[4-[9,9,18,18-tetrakis(2-ethylhexyl)-15-[7-[(E)-(3-ethyl-4-oxo-2-sulfanylidene-1,3-thiazolidin-5-ylidene)methyl]-2,1,3-benzothiadiazol-4-yl]-5,14-dithiapentacyclo[10.6.0.03,10.04,8.013,17]octadeca-1(12),2,4(8),6,10,13(17),15-heptaen-6-yl]-2,1,3-benzothiadiazol-7-yl]methylidene]-1,3-thiazolidin-4-one*


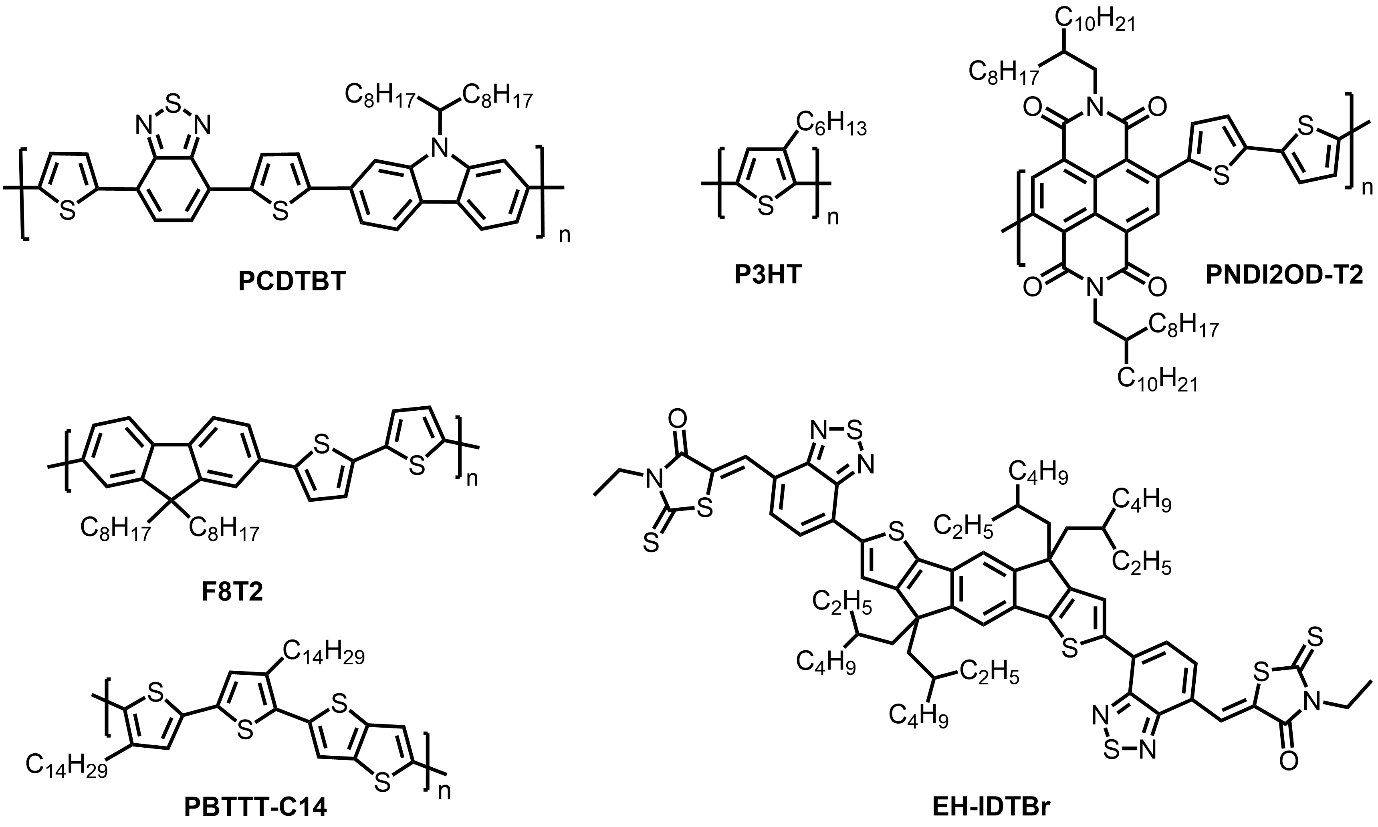


**Figure S1**. Chemical structures of investigated materials.

**Table S1.** Polymer batch information.

| **1-Material** | **Lot #** |
| --- | --- |
| PBTTT-C14 | YY26020CB |
| EH-IDTBr | - |

**Table S2.** Polymer molecular weights and dispersity.

| **Material** | **M_N_ (kDa)** | **PDI** |
| --- | --- | --- |
| F8T2 | 71 | 2.3 |
| PCDTBT | 40 | 2.2 |
| P3HT | 54 | 2.4 |
| PNDI2OD-T2 | 43 | 2.8 |


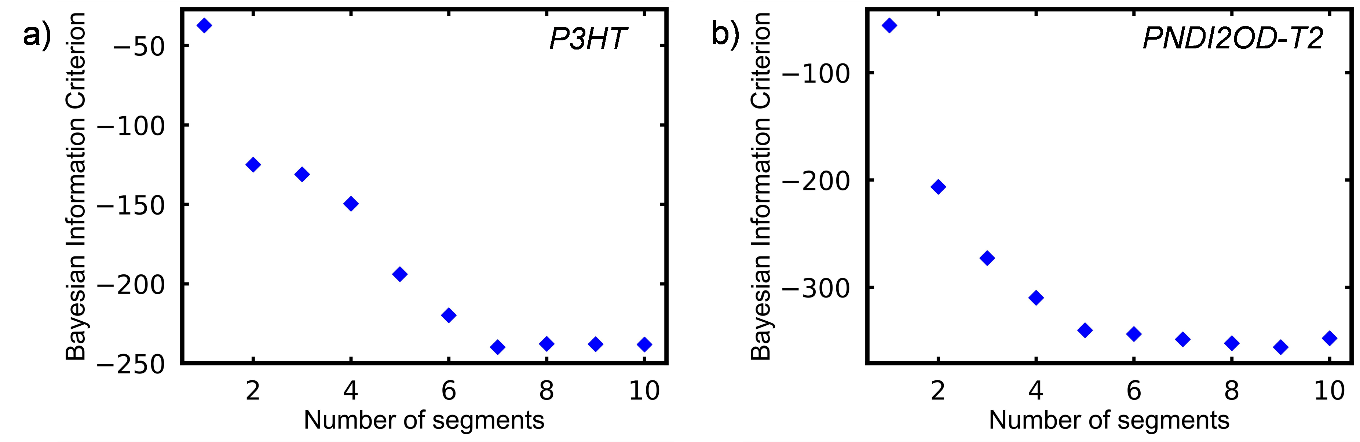


**Figure S2**. a, b) BIC calculation for SL-NDM in P3HT and PNDI2OD-T2 films, respectively.

**S2. Correlation between deviation metric and electronic coupling strength**

**Proof of concept: Weakly interacting H-aggregate polymer P3HT**

The normalized UV–vis of P3HT and corresponding NDM are shown in **Figure 5a,b.** The film absorption spectrum of P3HT is composed of two parts, a longer wavelength region, dominated by ordered structure regions of the film associated with weak interacting H-aggregate states, and a shorter wavelength region associated with more disordered molecular states (**Figure S3a**).^[1]^ The spectra at 250 °C is chosen as the spectra of the disorder region. We used the linear absorption spectroscopy method to estimate the spectra of disordered chains at each temperature and subtract to acquire the absorption spectrum of aggregates from temperature dependent in-situ spectra of P3HT,^[2]^ with representative examples shown in **Figure S3b-d**. To quantify the competition on the evolution of aggregated and amorphous states with increasing temperature, the area ratio of the aggregated region versus the amorphous region (Area_aggregate_/Area_amorphous_) and the absorbance ratio of aggregated region versus amorphous region (A_aggregate_/A_amorphous_) were calculated, as displayed in **Figure S3e,f**, respectively. The A_aggregate_/A_amorphous_ ratio decreases with increasing temperature and approach 0 above 230 °C, demonstrating the films are fully in an amorphous state. We observed the changing trend in A_aggregate_/A_amorphous_ ratio is reversely proportional to the change in NDM. This observation suggests that DM has a strong correlation with the changes in inter-molecular coupling strengths between chromophores. The automated PLR analysis is performed on A_aggregate_/A_amorphous_ ratio and SL-NDM plot, and similar thermal transition temperatures are observed.


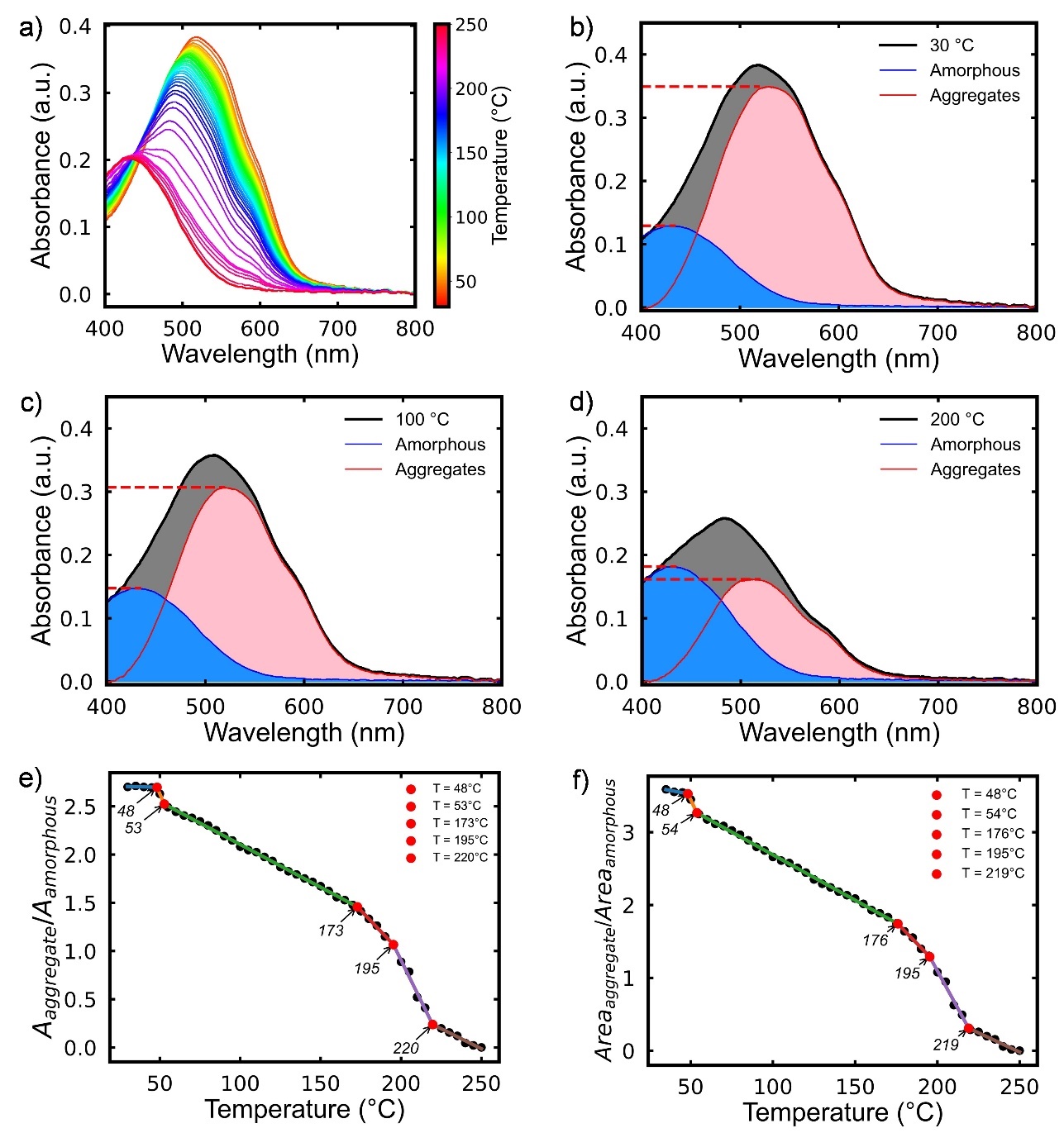


**Figure S3**. a) Temperature-dependent absolute absorbance of P3HT films. b-d) Representative examples to extract the absorbance spectra of aggregated and amorphous regions at 30, 100 and 250 °C, respectively. e) PLR analysis on the absorbance ratios of the aggregated spectra to the amorphous spectra. f) PLR analysis on area ratio of the integrated areas of aggregated region to the integrated areas amorphous region.

**S3. In-situ versus ex-situ UV**−**vis measurement**

In our work, we have successfully demonstrated the capability of using in-situ UV−vis in determining thermal transitions in conjugated materials thin films. Our in-situ UV−vis introduced here have two key differences in terms of experiment and data analysis in comparison to the method proposed by Root et al.^[3]^ Experimentally, the ex-situ UV−vis measurement involved to thermally anneal thin film to a specific temperature followed by cooling method to room temperature, and then the UV−vis spectra of thermally annealed films were recorded at the same room temperature. The ex-situ UV−vis measurement needs a large number of thin films that carries the risk of microstructural differences between films due to reproducibility issues in film casting dynamics and variation in probed regions. In contrast, the in-situ UV−vis monitors the evolution of the spectra with temperature in the same spot.^[4, 5]^

In ex-situ UV−vis measurement, the *T_g_* is estimated from the intersection of two linear regions in the linear-linear plot of DM versus temperature. Taking in-situ PCDTBT film as an example, there is likely no well-defined linear region at low temperature regime in linear-linear plot of DM versus temperature in our in-situ data (see **Figure S4**). Focusing on the selected temperature regions in PCDTBT where *T_g_* could be located as an example, the estimated transition from the intersection of two linear regions in the linear-linear plot is around 146 °C, largely deviating from its actual *T_g_* of 133 °C from DSC and DMA determined from semi-logarithmic plot (see **Figure S5**). The difference in experimentation and data analysis could be the reasons accounting for success in measuring the *T_g_* in PCDTBT with our method, in contrast to previous work.^[3]^ Additionally, various types of thermal transitions are effectively determined from semi-logarithmic plot, rather than limited to solely *T_g_*.


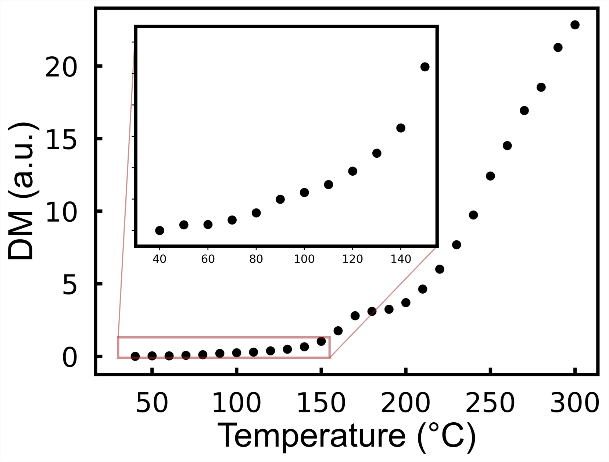


**Figure S4**. Enlarged selected low temperature regime of the deviation metric (DM) of PCDTBT film in a linear-linear plots.


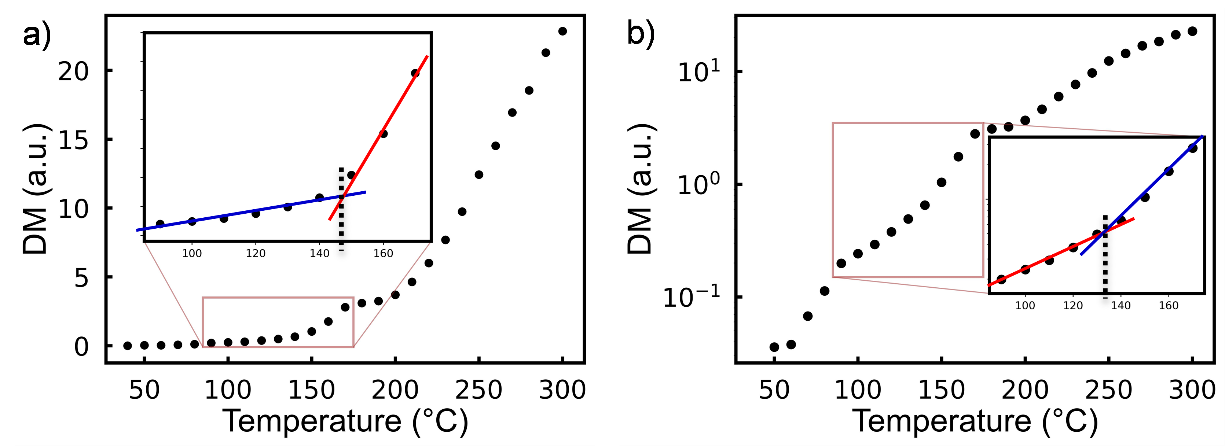


**Figure S5**. Comparison of a thermal transition determined from a) linear-linear plot. b) Semi-logarithmic plot in a PCDTBT film.


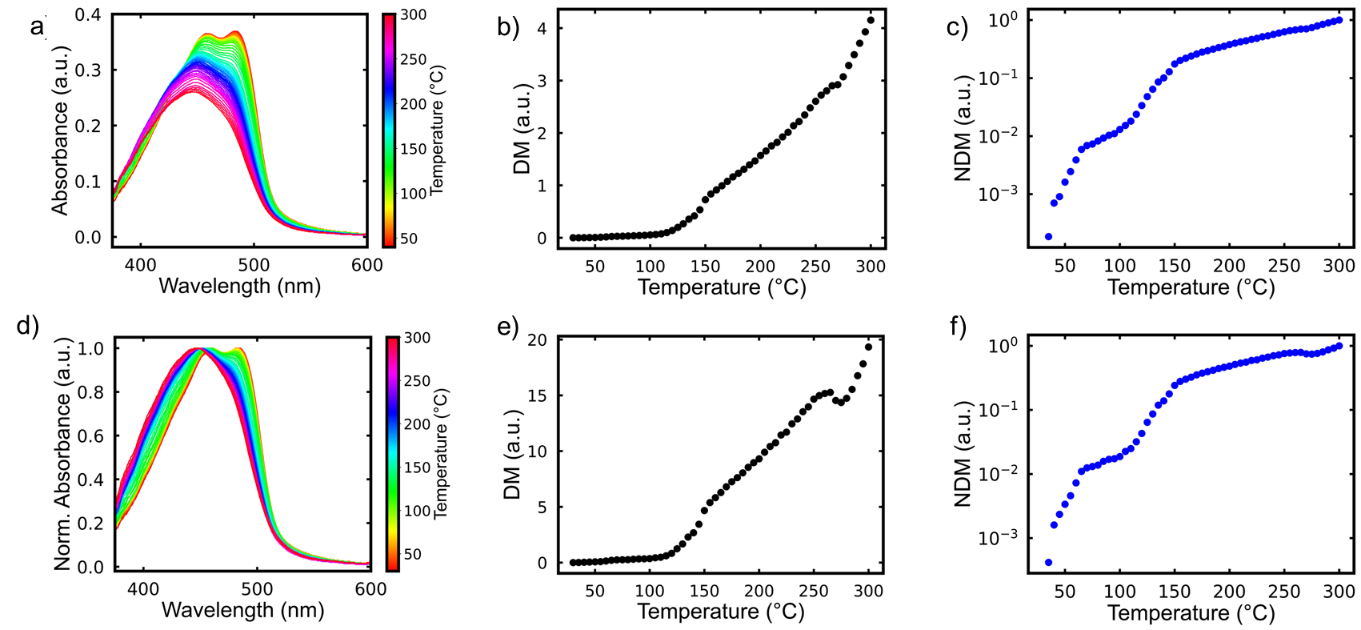


**Figure S6**. Comparison of deviation metrics calculated from in linear scale and semi-logarithmic plot in a F8T2 film. a-c) absolute absorbance; d-f) normalized absorbance.

**S4. Additional data and analysis**

**Table S3**. Comparison of thermal transitions in PCDTBT films with different characterization tools. Proposed identification of the transition is given at the bottom of the table.

| **Methods** | ***Sub-melting transition T* (°C)** | | | | ***T*** **(°C)** |
| --- | --- | --- | --- | --- | --- |
| NDM | 91 | 138 | 171 | 196 | 255 |
| λ_1_ | - | 143 | 175 | 204 | 241/253 |
| λ_2_ | 92 | 138 | 166 | 207 | 262 |
| DSC | 85 | 134 | 170 | - | 244 |
| DMA | - | 129 | 177 | 177-210 | - |
|  |  | *T_g_* |  |  | *T_iso_* |


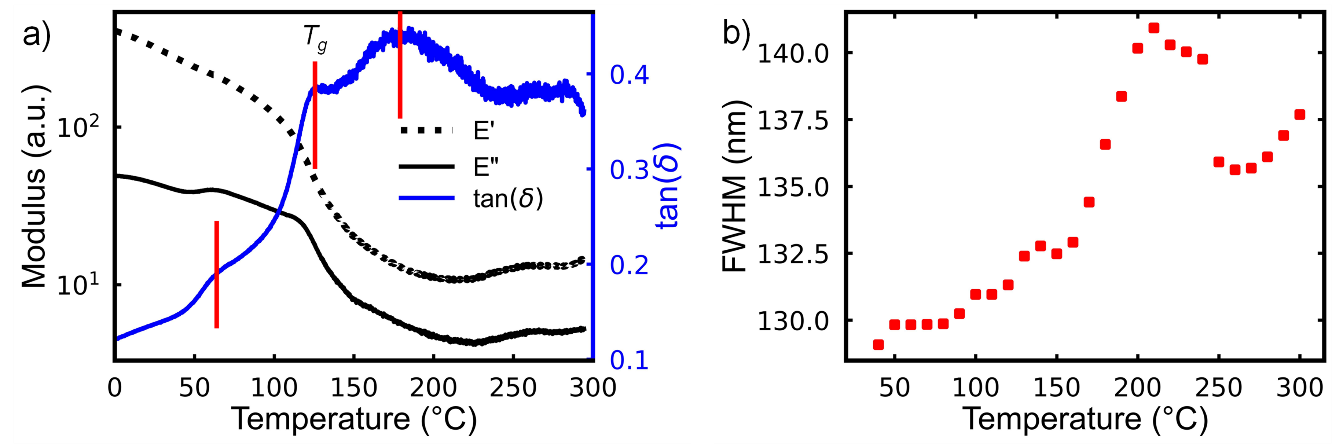


**Figure S7**. a) DMA scan with storage, loss modulus and tan δ of drop-cast PCDTBT film. b) Temperature-dependent FWHM plot of PCDTBT film extracted from temperature-dependent UV−vis spectra.

**Table S4**. Comparison of thermal transitions in F8T2 films with different characterization tools.

| **Methods** | ***Sub-melting transition T* (°C)** | | | | | | ***T* (°C)** |
| --- | --- | --- | --- | --- | --- | --- | --- |
| NDM | 65 | 111 | 132 | 157 | 252 | 279 |  |
| λ_1_ | 65 | 113 | - | 153 | - | - |  |
| λ_2_ |  | 112 | 142 | 154 | 262 | 278 |  |
| DSC | - | 108 | 122-140 | - | 251^#^ | 276 | 312 |
| DMA | 64 | 105 | 131 | - | - | 280 |  |
|  |  | *T_g_* | *T_cc_* |  |  |  | *T_iso_* |

^#^Extracted from the 2^nd^ heat cycle from DSC.


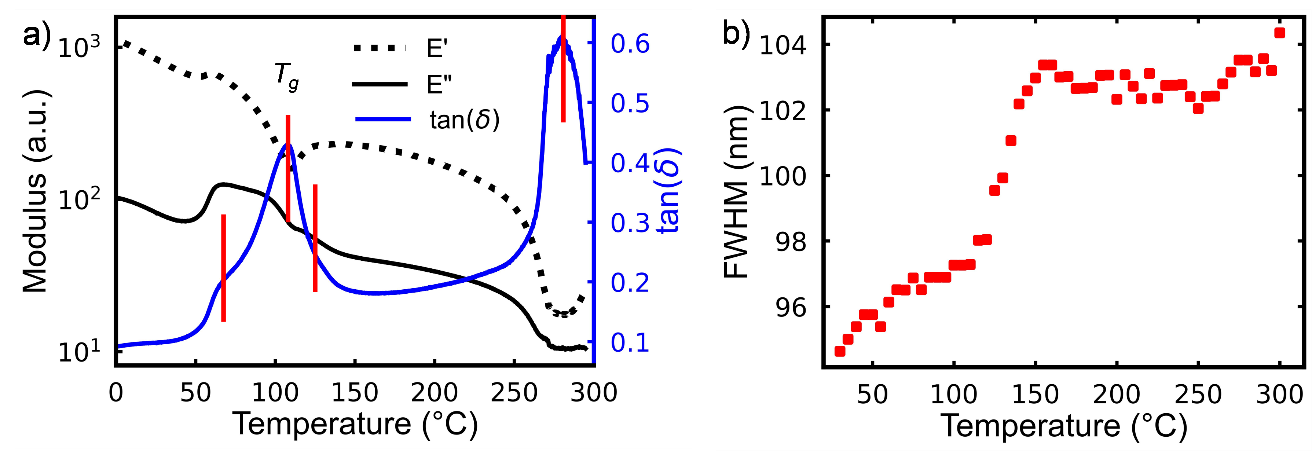


**Figure S8**. a) DMA scan with storage, loss modulus and tan δ of drop-cast F8T2 film. b) Temperature-dependent FWHM plot of F8T2 film extracted from temperature-dependent UV−vis spectra.

**Table S5**. Comparison of thermal transitions in PBTTT films with different characterization tools.

| **Methods** | ***Sub-melting transition T* (°C)** | | | | ***T_m_* (°C)** |
| --- | --- | --- | --- | --- | --- |
| NDM | 87 | 117 | 167 | - | 237 |
| λ_1_ | **-** | - | 146 | 185 | 240 |
| λ_2_ | 66 | - | 174/175 | 204 | 248 |
| DSC | 84 | 117 | - | - | 205-240 |
| DMA |  | 116 | 144-160 | 204-229 | 229 |
|  |  | *T_lc_* |  |  | *T_m_* |


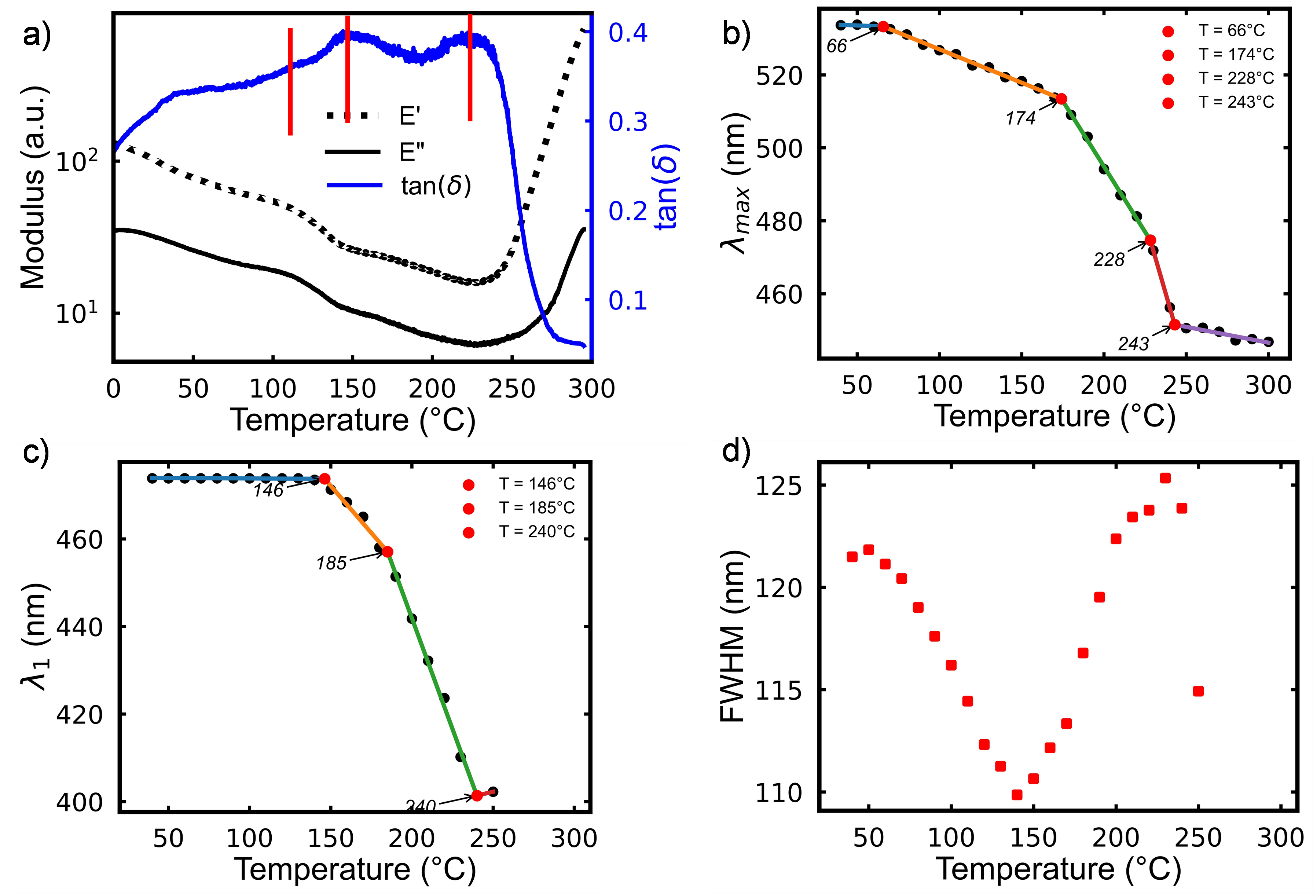


**Figure S9**. a) DMA scan with storage, loss modulus and tan δ of drop-cast PBTTT-C14 film. b-d) Temperature-dependent λ_max_, λ_1_ and FWHM plots of PBTTT-C14 film extracted from temperature-dependent UV−vis spectra, respectively.

**Table S6**. Comparison of thermal transitions in P3HT films with different characterization tools and reported literature.

| **Methods** | ***Sub-melting transition T* (°C)** | | | | ***T_m_* (°C)** | ***References*** |
| --- | --- | --- | --- | --- | --- | --- |
| NDM | 47 | 62 | 101 | 193 | 220 |  |
| λ_1_ | - | - | - | 187 | 215 |  |
| λ_2_ | - | - | - | 193 | 214-219 |  |
| DSC | 40-80 | | - | - | 220 |  |
| DMA | - | - | - | 195 | 230 |  |
| Literature | | | | | |  |
| SE | - | 71 | 117 | 196 | 228 | ^[6]^ |
| FSC | 50 | - | - | 180 | 220 | ^[7]^ |
| DMA | 25 | - | 100 | - | 230 | ^[8]^ |


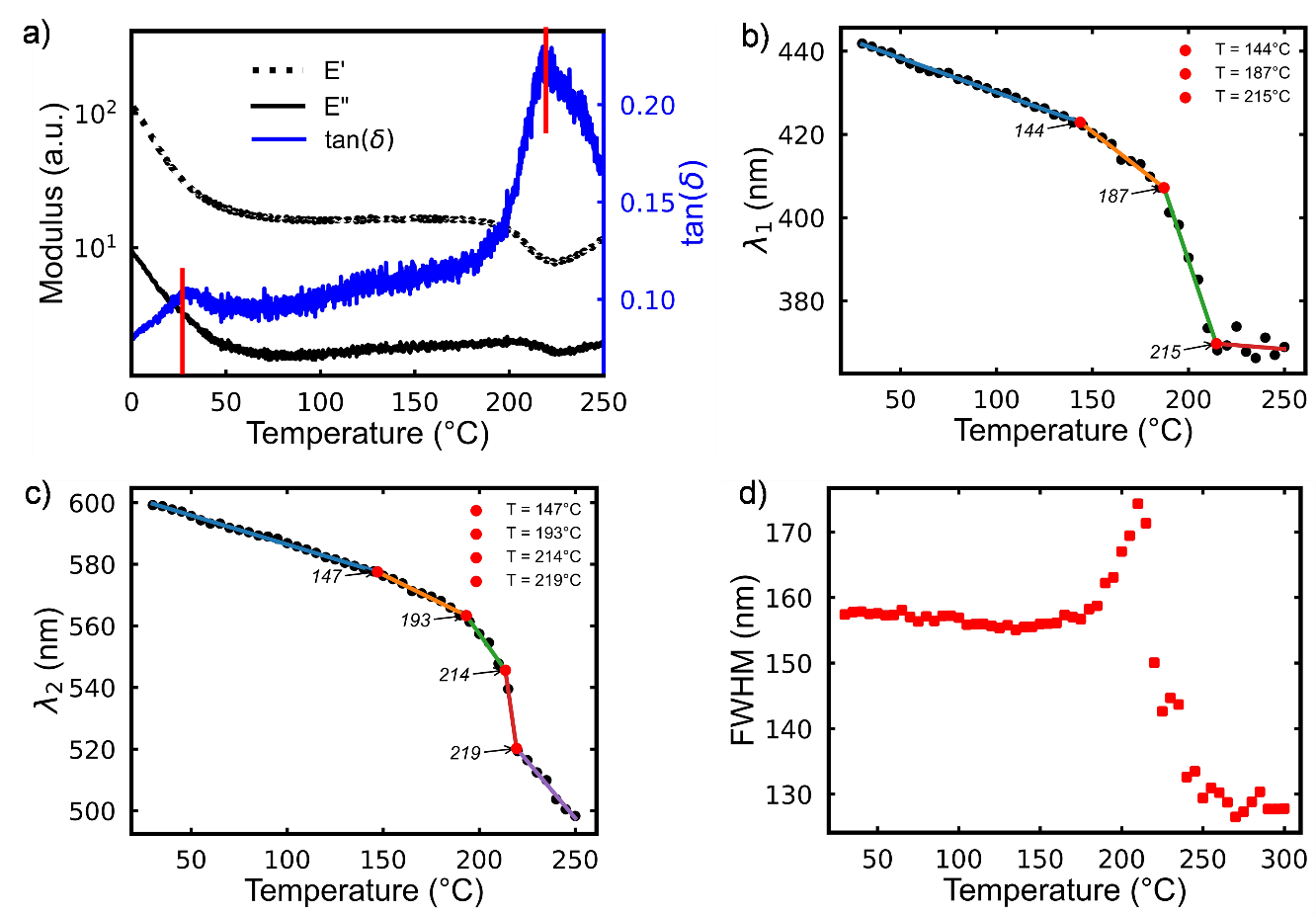


**Figure S10**. a) DMA scan with storage, loss modulus and tan δ of drop-cast P3HT film. b-d) Temperature-dependent λ_1_, λ_2_ and FWHM of P3HT extracted from in situ UV−vis spectra, respectively.

**Table S7**. Comparison of thermal transitions in PNDI2OD-T2 films with different characterization tools and reported literature.

| **Methods** | ***Sub-melting transition T* (°C)** | | | | | | ***T_m_* (°C)** | ***Reference*** |
| --- | --- | --- | --- | --- | --- | --- | --- | --- |
| NDM | 67 | 96 | 133 | 185 | 237 | - | - |  |
| λ_1_ | - | - | - | 194 | - | 270^#^ | - |  |
| λ_2_ | - | - | - | 171 | - | - | 296 |  |
| DSC | 60-110 | | - | - | 232 | - | 310 |  |
| DMA | 60-74 | - | - | - | - | 268^#^ | - |  |
| Literature | | | | | | | |  |
| SE | - | - | 120 | 180 | - | - | 310 | ^[6]^ |
|  |  |  |  |  | *T_ss_* |  | *T_m_* |  |


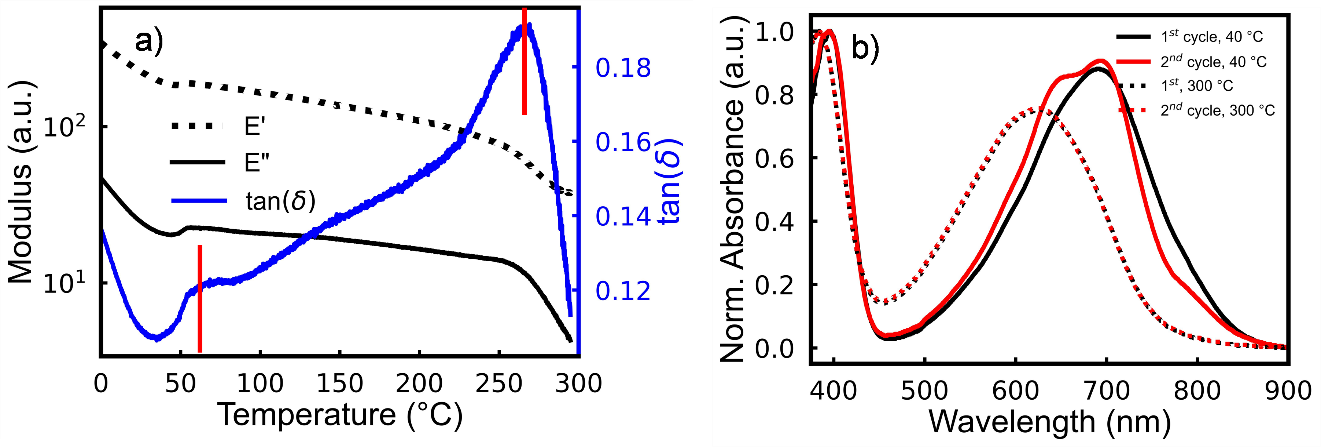


**Figure S11**. a) DMA scan with storage, loss modulus and tan δ of drop-cast PNDI2OD-T2 film. b) Spectral comparison between the first and the second cycle at 40 °C and 300 °C.


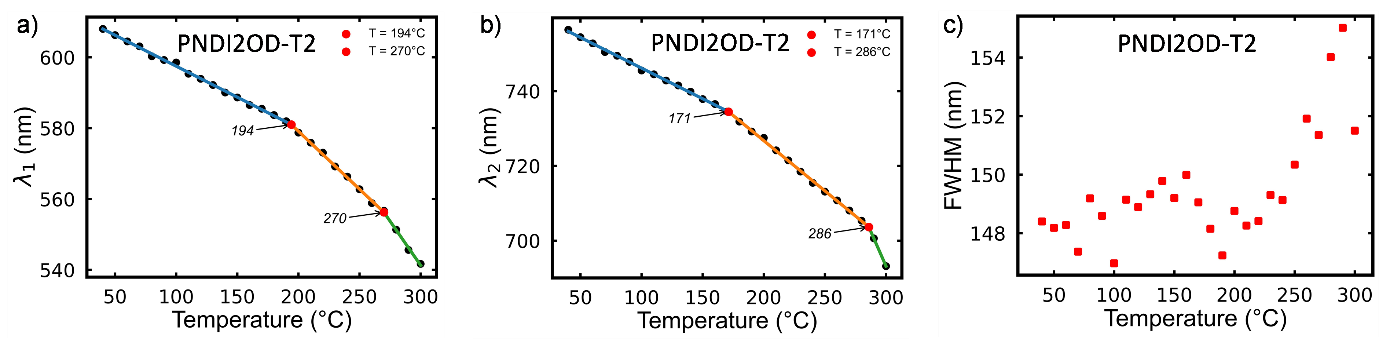
**Figure S12**. a-c) Temperature-dependent λ_1_, λ_2_ and FWHM of PNDI2OD-T2 extracted from in situ UV−vis spectra, respectively.

**Table S8**. Comparison of thermal transitions in EH-IDTBr films with different characterization tools and reported literature.

| **Methods** | ***Sub-melting transition T* (°C)** | | | | | ***T_m1_* (°C)** | ***T_m2_* (°C)** |
| --- | --- | --- | --- | --- | --- | --- | --- |
| NDM | 67 | 104 | 119 | 146 | 173 | 182 | - |
| λ_1_ | - | 107 | 120 | 146 | 173 | 182 | 221 |
| λ_2_ | - | 107 | 121 | 153 | 173 | 187 | 220 |
| DSC | - | 106 | 117/128 | 150 | 170 | 179 | - |
|  |  | *T_g_* | *T_lc_* |  |  | *T_m1_* | *T_m2_* |


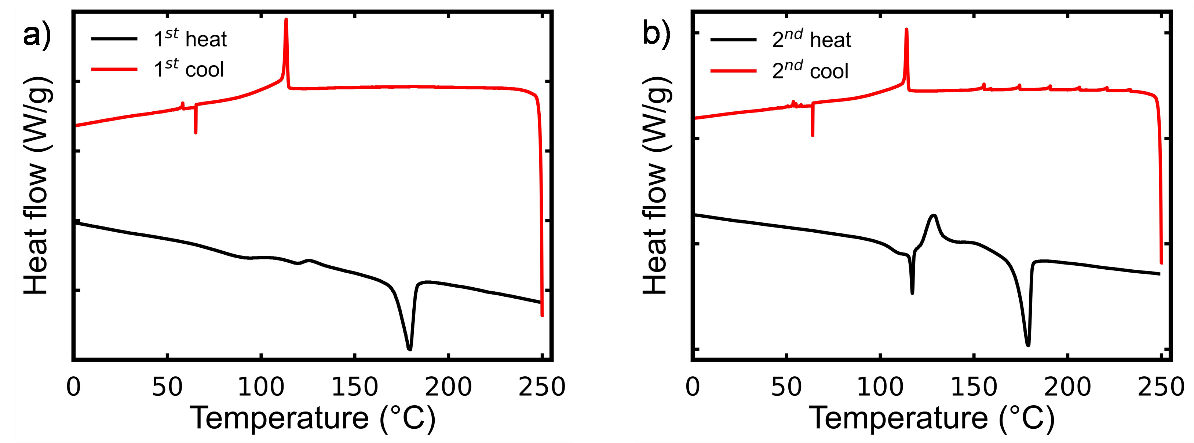


**Figure S13**. a-b) The 1^st^ and 2^nd^ heat DSC thermogram of EH-IDTBr.


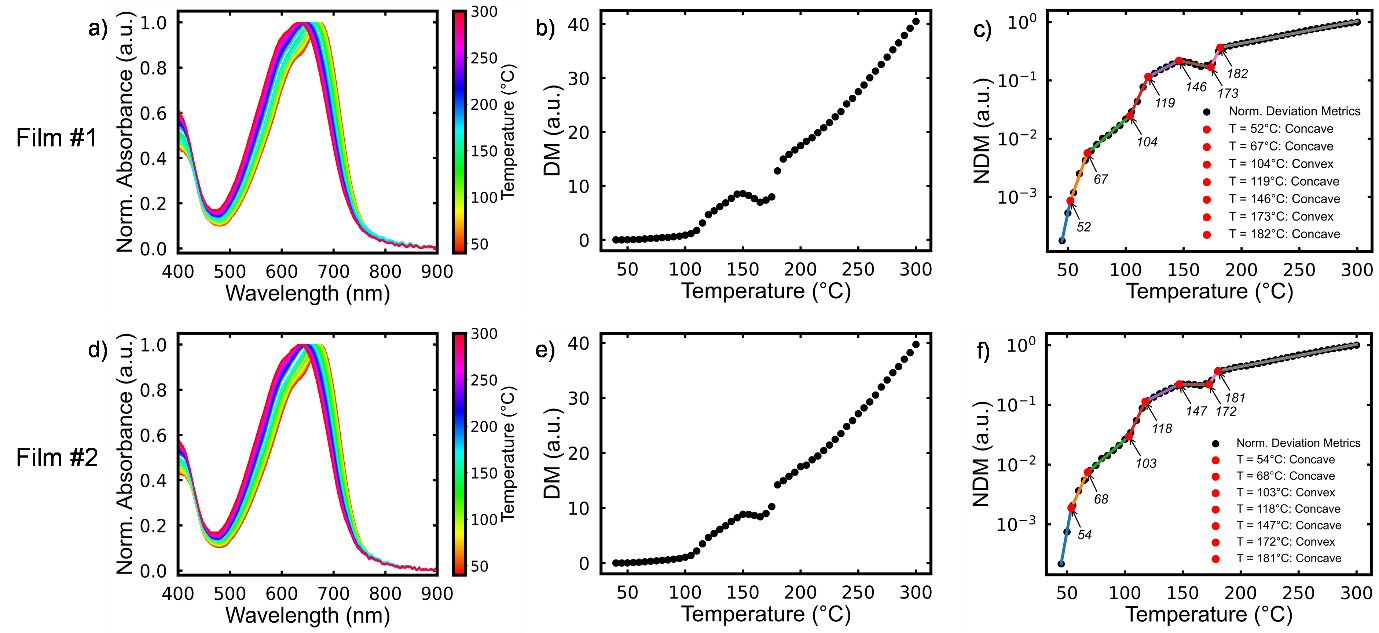
**Figure S14**. Comparison between temperature-dependent UV−vis, DM and SL-LSDs analysis between two separate EH-IDTBr films. a-c) Film #1. d-f) Film #2.


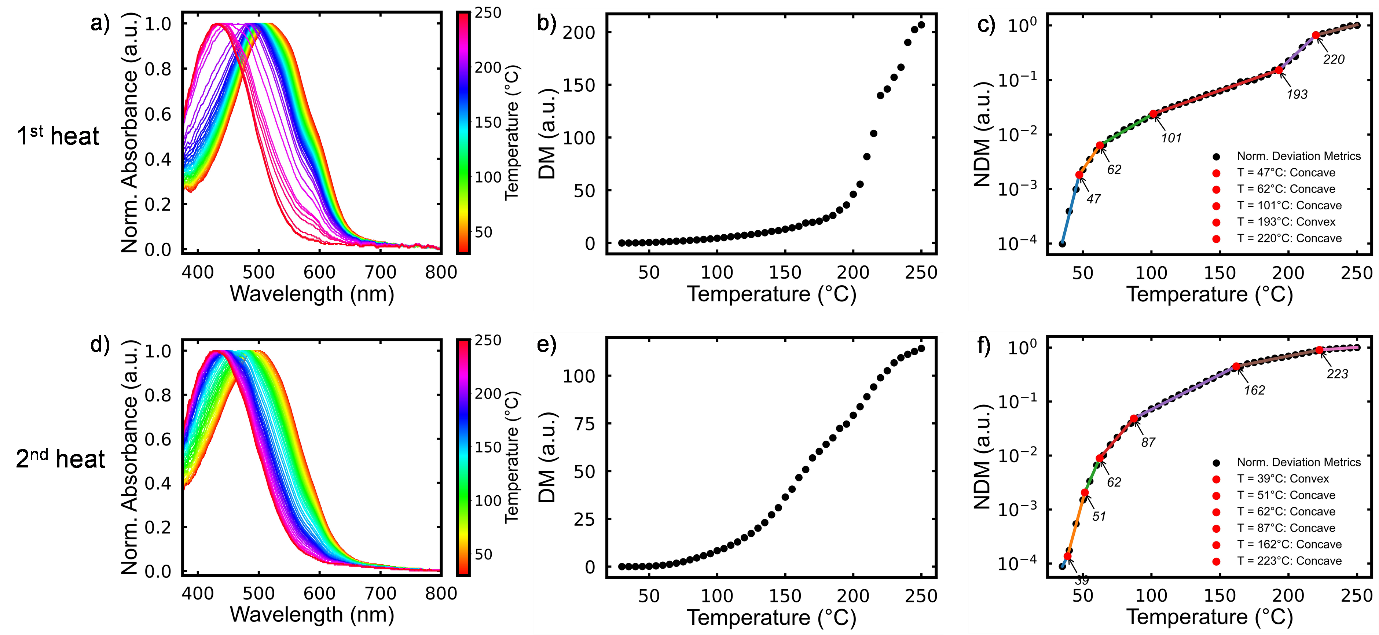
**Figure S15**. Comparison between the 1^st^ and 2^nd^ heat in P3HT films: a-c) Temperature-dependent UV−vis, DM and SL-LSDs of the 1^st^ heat P3HT, respectively. d-f) Temperature-dependent UV−vis, DM and SL-LSDs of the 2^nd^ heat P3HT, respectively.


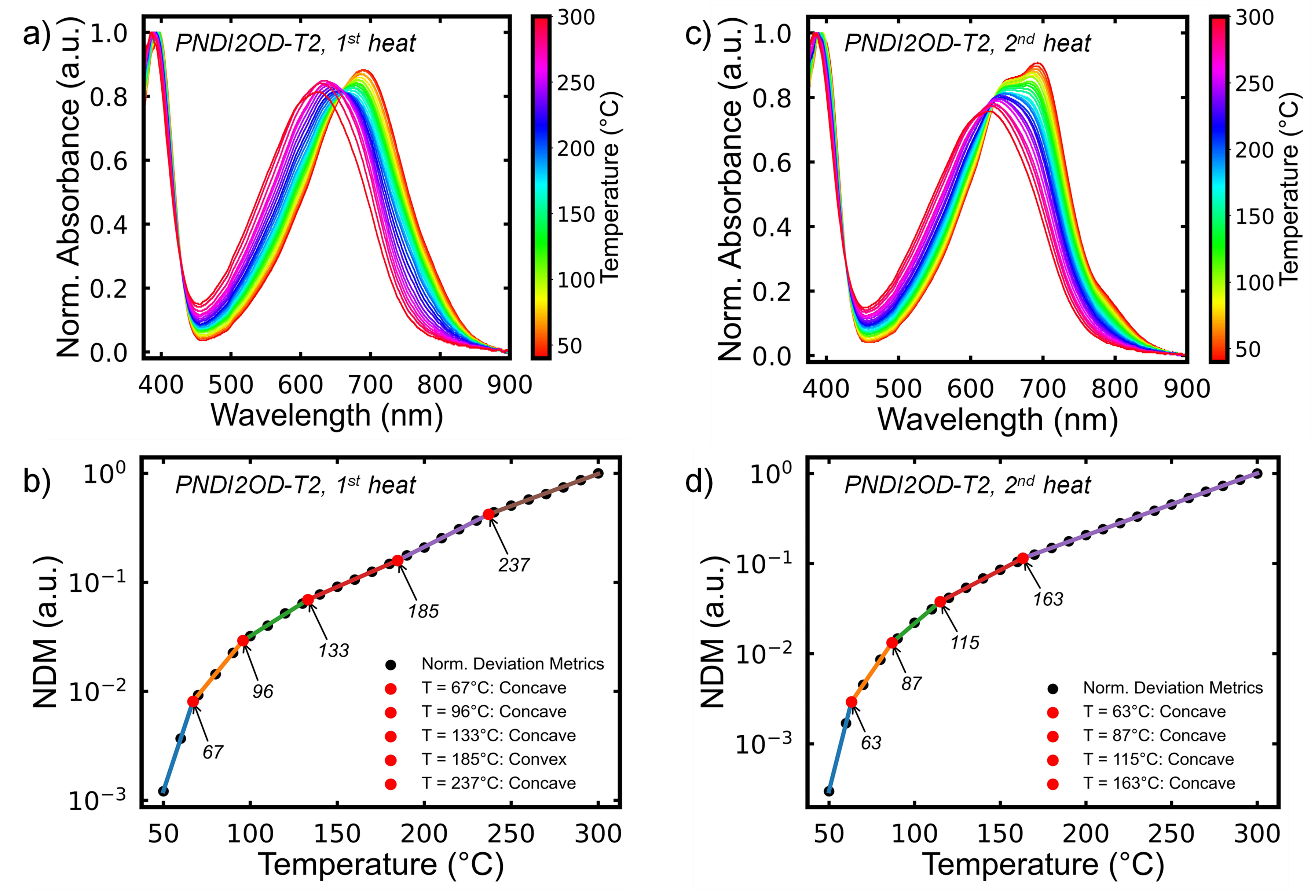


**Figure S16**. Comparison between the 1^st^ and 2^nd^ heat in PNDI2OD-T2 films: a, b) Temperature-dependent UV−vis and SL-LSDs of the 1^st^ heat, respectively. c, d) Temperature-dependent UV−vis and SL-LSDs of the 2^nd^ heat, respectively.


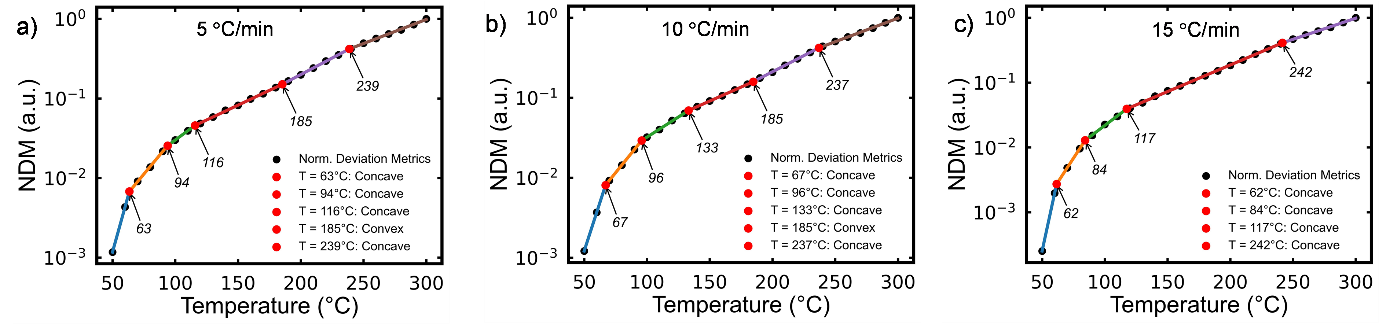
**Figure S17**. SL-LSDs analysis to determine thermal transitions of the in PNDI2OD-T2 films at different heating rates: a-c) ∼15 °C/min, ∼10 °C/min, and ∼5 °C/min, respectively.


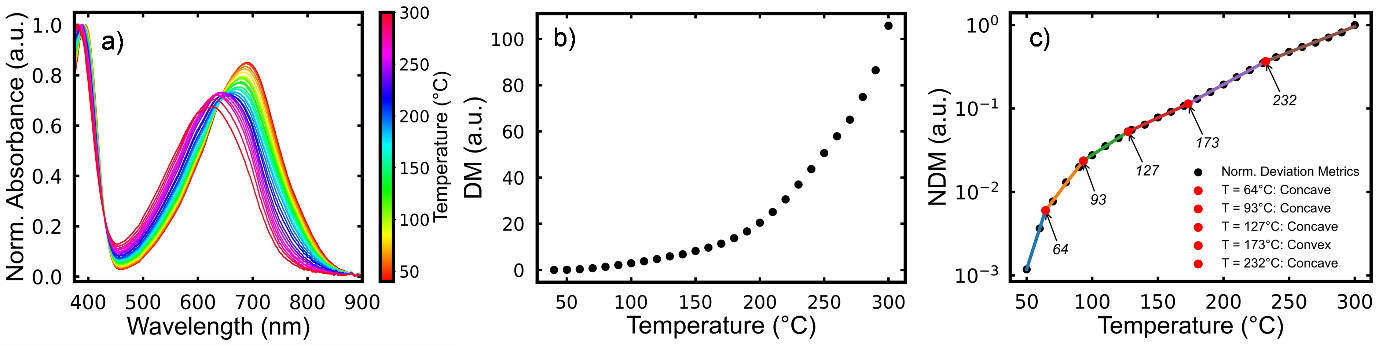


**Figure S18**.  a-c) Temperature-dependent UV−vis, DM plot, and the SL-LSDs analysis to detect thermal transitions of the first heat, respectively, in PNDI2OD-T2 film, with 2-min isotherm for each temperature.

The impact of collected temperature interval (T-interval) and compared the variation in SL-LSD-determined transition temperatures in two systems, EH-IDTBr and PNDI2OD-T2. A direct parallel comparison between determined thermal transitions in EH-IDTBr film is shown in **Figure S19,** **Table S9** and in PNDI2OD-T2 is shown in **Figure S20**. Indeed, there is a very small discrepancy in the probed transitions between 10 °C and 5 °C intervals.


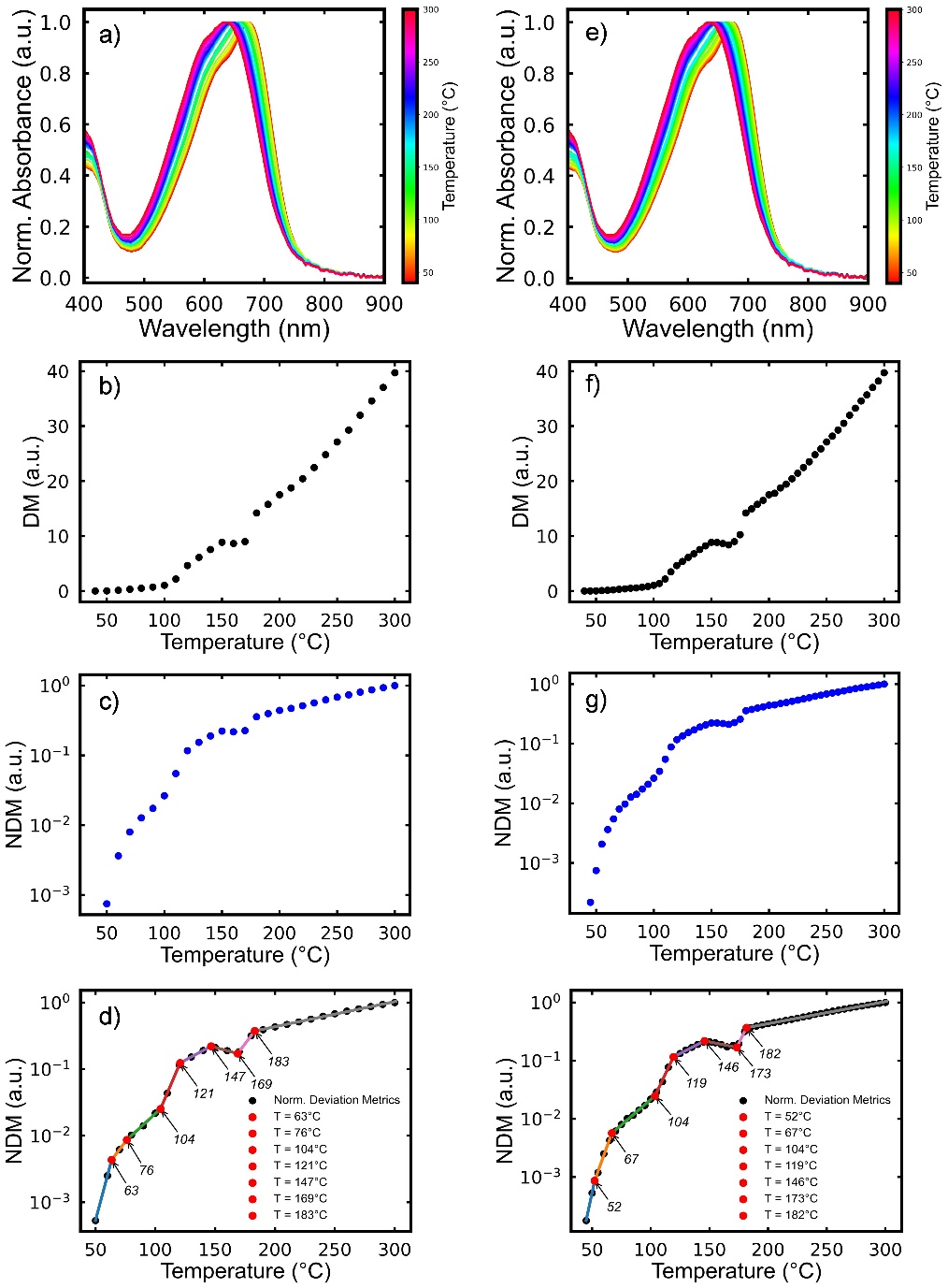


**Figure S19**. a-d) and e-h) Temperature-dependent UV−vis spectra, deviation metrics, normalized deviation metrics and the SL-LSDs analysis to detect thermal transitions in EH-IDTBr film, respectively, with 10 °C interval and 5 °C interval.


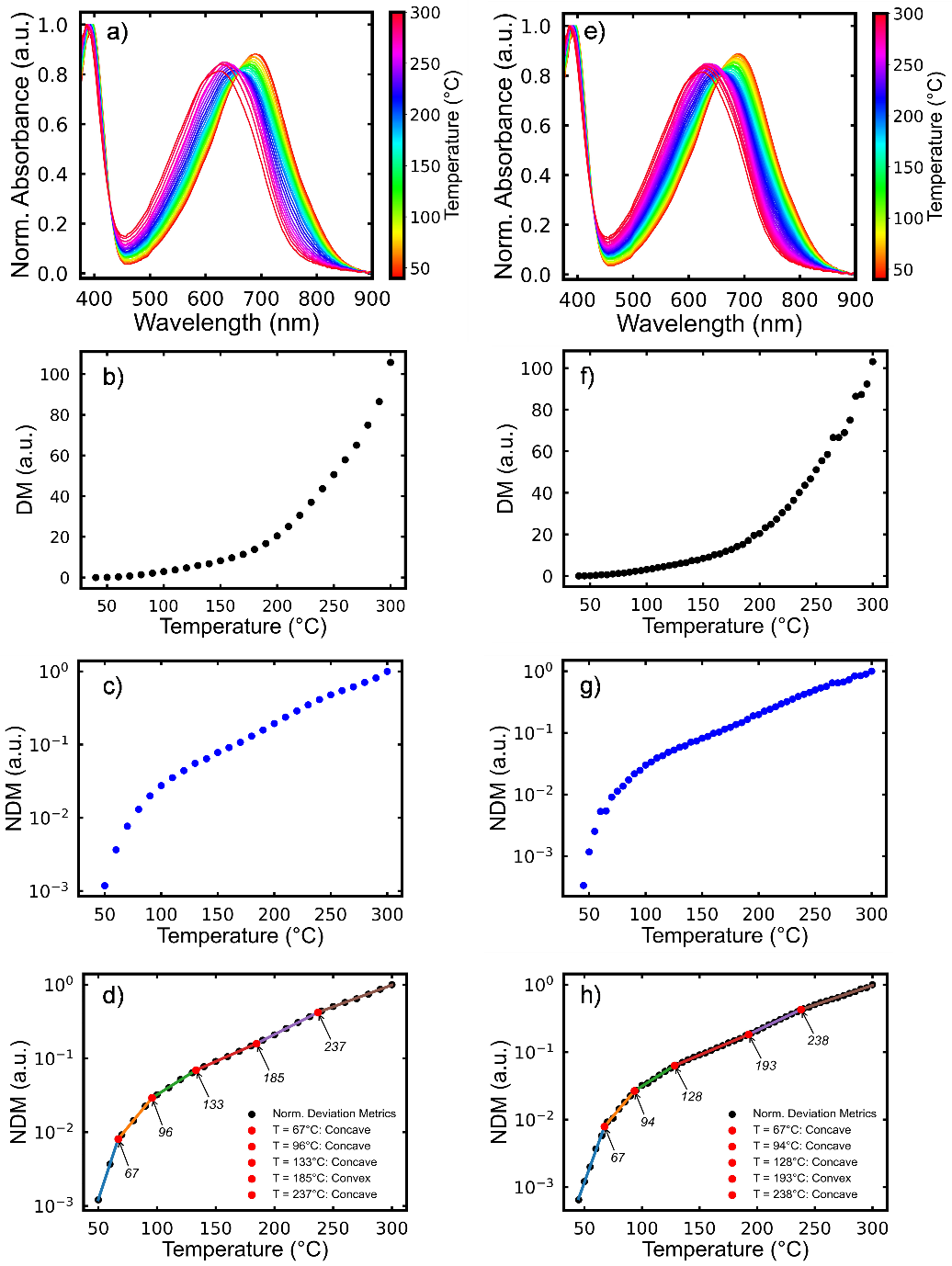


**Figure S20**. a-d) and e-h) Temperature-dependent UV−vis spectra, deviation metrics, normalized deviation metrics and the SL-LSDs analysis to detect thermal transitions in PNDI2OD-T2 film, respectively, with 10 °C interval and 5 °C interval.

**Table S9**. Comparison of thermal transitions in EH-IDTBr films with different T-intervals and tools.

| **Experiments** | **Low temperature transition (°C)** | ***T_g_* (°C)** | ***T_lc_* (°C)** | ***Other transitions***  **(°C)** | ***T_m_* (°C)** |
| --- | --- | --- | --- | --- | --- |
| UV−vis, Film #1, 10°C interval | 63, 76 | 104 | 121 | 147, 169 | 183 |
| UV−vis, Film #1, 5°C interval | 52, 67 | 104 | 119 | 146, 173 | 182 |
| UV−vis, Film #2, 5°C interval | 54, 68 | 103 | 118 | 147, 172 | 181 |
| DSC | N/A | 106 | 117 | 150, 170 | 179 |

N/A: Too subtle to be detected from the analysis.

**S5. Temperature calibration of stage and film**

To evaluate potential systematic temperature deviations, we compared the nominal Linkam stage temperature (T_LK_) and the temperature measured directly on the polymer thin film surface (𝑇_S_) over range of 30–250 °C using the same heating rate with ATLAS measurement. Linear regression of the calibration data yields:

$$T_{S} 0.985T_{LK}+0.55$$

with R^2^ >0.999.

The calibration data and regression fit are shown in **Figure S21**. The maximum deviation over the investigated temperature range (30–250 °C) is within ±4 °C. We note that all ATLAS measurements were performed with the Linkam stage fully enclosed and under vacuum. Under these conditions, convective heat loss from the film surface is suppressed, and heat transfer is dominated by conduction from the stage through the glass substrate.

However, the thermocouple calibration measurements were performed with the stage partially open and without vacuum. In this measurement, natural convection from the exposed film surface contributes to additional heat dissipation. Because the convective heat transfer coefficient increases with temperature, this effect becomes more pronounced at higher nominal stage temperatures. This behavior is consistent with the slightly lower measured surface temperature ($T_{S}$) relative to the nominal stage temperature ($T_{LK}$) at elevated temperatures.

Therefore, the small systematic deviation observed in the open-stage thermocouple calibration likely represents a conservative upper bound of the thermal offset. Under the actual vacuum conditions used in ATLAS experiments, convective losses are minimized, and the film temperature is expected to more closely match the stage temperature. Applying the linear correction does not alter the assigned transition temperatures within experimental uncertainty and does not affect the conclusions of this work.


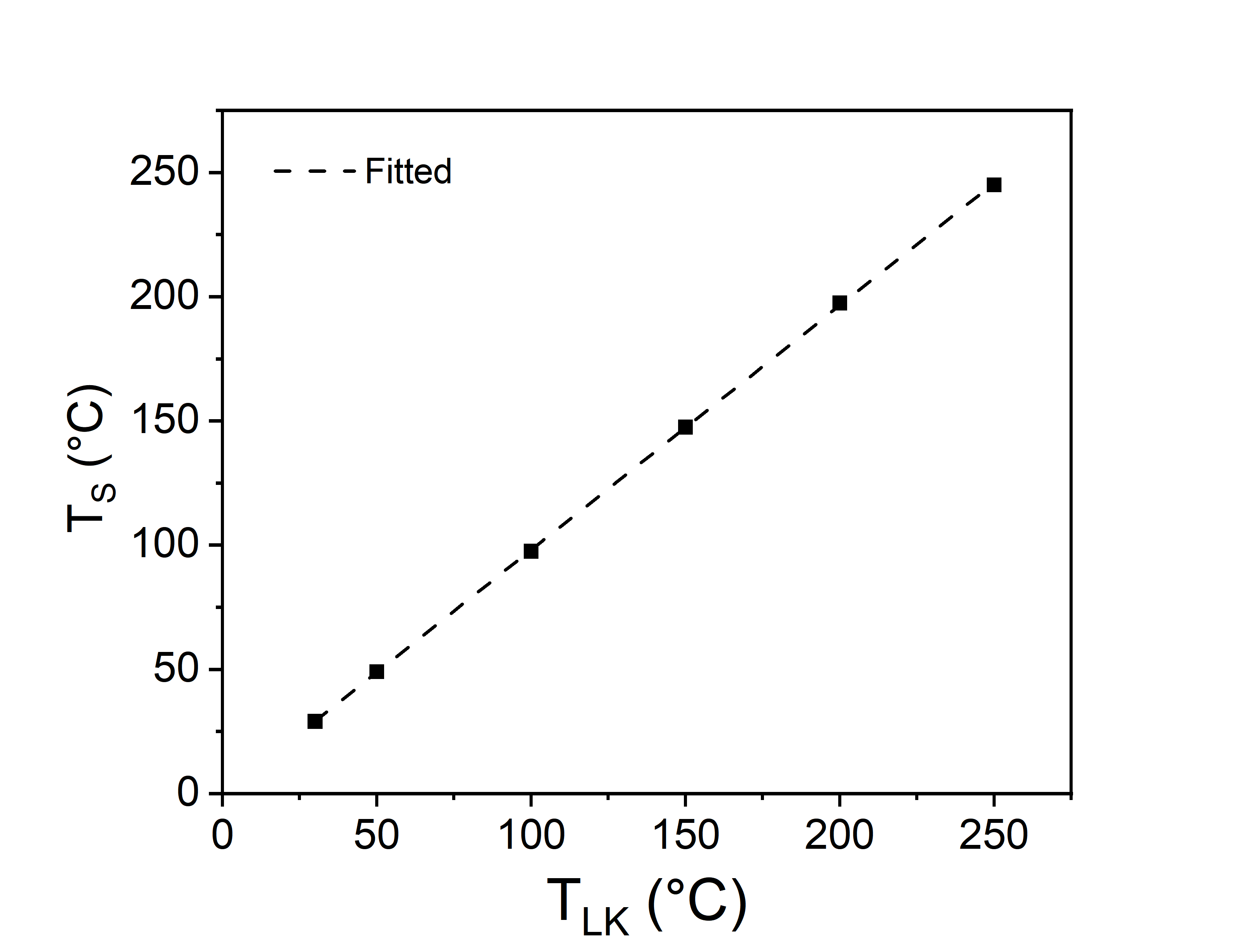


**Figure S21**. Linear relation between the Linkam stage temperature (T_LK_) and the polymer thin film temperature, 𝑇_S_.

**References**

1 Clark, J.; Silva, C.; Friend, R. H.; Spano, F. C. Role of Intermolecular Coupling in the Photophysics of Disordered Organic Semiconductors: Aggregate Emission in Regioregular Polythiophene. *Phys. Rev. Lett.* 2007, ***98***, 206406.

2 Clark, J.; Chang, J.-F.; Spano, F. C.; Friend, R. H.; Silva, C. Determining exciton bandwidth and film microstructure in polythiophene films using linear absorption spectroscopy. *Appl. Phys. Lett.* 2009, ***94***.

3 Root, S. E.; Alkhadra, M. A.; Rodriquez, D.; Printz, A. D.; Lipomi, D. J. Measuring the Glass Transition Temperature of Conjugated Polymer Films with Ultraviolet–Visible Spectroscopy. *Chem. Mater.* 2017, ***29***, 2646-2654.

4 Pingel, P.; Zen, A.; Abellón, R. D.; Grozema, F. C.; Siebbeles, L. D. A.; Neher, D. Temperature-Resolved Local and Macroscopic Charge Carrier Transport in Thin P3HT Layers. *Adv. Funct. Mater.* 2010, ***20***, 2286-2295.

5 Schrickx, H. M.; Moore, C.; Shafe, A. A.; Samadani, A.; Balar, N.; Vu, D.; Rech, J. J.; You, W.; O’Connor, B. T. Discerning Thermal Transition Behavior of Conjugated Polymers through In Situ Optical Characterization of Oriented Films. *Chem. Mater.* 2024, ***36***, 11119-11128.

6 Henry, R.; Balar, N.; Ade, H. In-Situ Ellipsometry for the Determination of Thermal Transitions and Relaxations in Organic Photovoltaic Materials. *Chem. Mater.* 2023, ***35***, 7406-7421.

7 Martín, J.; Stingelin, N.; Cangialosi, D. Direct Calorimetric Observation of the Rigid Amorphous Fraction in a Semiconducting Polymer. *J. Phys. Chem. Lett.* 2018, ***9***, 990-995.

8 Balar, N.; Siddika, S.; Kashani, S.; Peng, Z.; Rech, J. J.; Ye, L.; You, W.; Ade, H.; O’Connor, B. T. Role of Secondary Thermal Relaxations in Conjugated Polymer Film Toughness. *Chem. Mater.* 2020, ***32***, 6540-6549.
